# Proteoglycans as Molecular Regulators of Bone Metastasis: Extracellular Matrix Remodeling, Tumor–Bone Crosstalk, Dormancy, and Therapeutic Opportunities

**DOI:** 10.3390/biom16071039

**Published:** 2026-07-16

**Authors:** Zoila Mora Guzmán, Ibzan Jahzeel Salvador Ibarra, Patricia Juárez, Anahí Jobeth Borrás Enríquez, Edmar de Jésús Díaz García, Hector Alejandro Cabrera-Fuentes, María Teresa Hernández-Huerta

**Affiliations:** 1 Secretaría de Ciencia, Humanidades, Tecnología e Innovación (SECIHTI), Facultad de Medicina y Cirugía, Universidad Autónoma “Benito Juárez” de Oaxaca, Oaxaca 68020, Mexico; zmora.fmc@uabjo.mx; 2 Centro de Investigación Facultad de Medicina UNAM-UABJO, Facultad de Medicina y Cirugía, Universidad Autónoma “Benito Juárez” de Oaxaca, Oaxaca 68020, Mexico; edg.89@live.com.mx; 3 Instituto Mexicano del Seguro Social, Hospital General de Zona con Medicina Familiar No. 18, Torreón 27220, Mexico; ibzjah@gmail.com; 4 Biomedical Innovation Department, Center for Scientific Research and Higher Education at Ensenada, Ensenada 22860, Mexico; pjuarez@cicese.mx; 5 División de Estudios de Posgrado e Investigación, Tecnológico Nacional de México/Instituto Tecnológico de Tuxtla Gutiérrez, Tuxtla Gutiérrez 29050, Mexico; anajoborras@gmail.com; 6 R&D Group, Vice Presidency for Scientific Research and Innovation, Imam Abdulrahman Bin Faisal University, P.O. Box 1982, Dammam 31441, Saudi Arabia; 7 División de Estudios de Posgrado e Investigación, Tecnológico Nacional de México/Instituto Tecnológico de Tijuana, Tijuana 22414, Mexico

**Keywords:** proteoglycans, bone metastasis, extracellular matrix, breast cancer, prostate cancer, perlecan, versican, decorin, hyaluronan, metastatic niche

## Abstract

**Background**: Bone metastasis is a frequent and debilitating complication of advanced cancer, particularly in breast and prostate cancer, and is driven by complex interactions among tumor cells, bone-resident cells, immune populations, vascular components, and the extracellular matrix. Within this specialized microenvironment, proteoglycans have emerged as key molecular regulators of tumor–bone crosstalk, matrix remodeling, metastatic niche formation, dormancy, and therapeutic resistance. **Methods**: We conducted a narrative review using targeted searches of PubMed and Google Scholar for studies published through 31 May 2026. Search terms included combinations of proteoglycan- and glycosaminoglycan-related concepts, including “proteoglycans,” “glycosaminoglycans,” “heparan sulfate proteoglycans,” “hyaluronan,” “heparanase,” “syndecans,” “glypicans,” “perlecan/HSPG2,” “versican,” and “decorin,” with disease- and process-related terms such as “bone metastasis,” “extracellular matrix,” “tumor–bone crosstalk,” “breast cancer,” “prostate cancer,” “metastatic niche,” “osteolytic metastasis,” “osteoblastic metastasis,” “dormancy,” “reactivation,” “immune regulation,” and “therapy resistance.” Original studies, reviews, and translational reports were selected according to their relevance to cell-surface, pericellular, and extracellular proteoglycans in bone metastatic progression. **Results**: Proteoglycans and associated GAG/ECM axes are implicated in multiple processes involved in skeletal metastasis, including growth factor availability, extracellular matrix organization, osteolytic and osteoblastic niche formation, angiogenesis, immune evasion, metastatic dormancy, reactivation, and therapy resistance. These functions are highly context-dependent and are influenced by proteoglycan localization, core protein structure, glycosaminoglycan composition, sulfation patterns, proteolytic processing, and cellular source. **Conclusions**: Proteoglycans represent critical molecular nodes in the bone metastatic microenvironment and hold potential as biomarkers, therapeutic targets, and tools for stratifying metastatic niche heterogeneity. Their clinical translation will require validation in human bone metastasis samples, improved models that reproduce the mineralized and immune-rich bone niche, and a clearer distinction between causal mechanisms and correlative associations. Future studies should integrate matrisome profiling, spatial proteomics, single-cell and spatial transcriptomics, glycosaminoglycan omics, degradomics, and three-dimensional bone niche models to define actionable proteoglycan-dependent mechanisms and improve therapeutic targeting of metastatic bone disease.

## 1. Introduction

Bone metastasis (BM) represents one of the most devastating complications of advanced cancer and remains a major cause of morbidity and mortality worldwide [1,2]. BM occurs frequently in breast cancer, prostate cancer (PCa), and lung cancer, in which breast cancer and PCa account for more than 80% of cases [3]. Despite advances in early diagnosis and systemic therapies, bone tumor dissemination continues to be associated with skeletal-related events (SREs) such as pathological fractures, severe pain, spinal cord compression, hypercalcemia, impaired quality of life, and treatment resistance [4,5]. Breast and prostate cancers exhibit a marked tropism for bone tissue, while lung, kidney, and thyroid cancers, as well as multiple myeloma, also show a high frequency of skeletal involvement [2,6,7]. Importantly, metastasis, rather than the primary tumor itself, is responsible for the majority of cancer-related deaths, underscoring the need to understand the molecular mechanisms that regulate metastatic colonization and its progression [7,8].

The establishment of BM is a dynamic, multi-stage process involving cell detachment from the primary tumor, epithelial–mesenchymal transition, invasion of the extracellular matrix, intravasation, survival in circulation, immune evasion, extravasation, latency/dormancy, and colonization of distant organs [9,10]. Among the various metastatic organs, bone provides a particularly permissive microenvironment, enriched with growth factors, cytokines, chemokines, mineralized matrix proteins, and specialized cell populations that promote tumor survival and expansion [11]. Osteoblasts, osteoclasts, osteocytes, bone marrow stromal cells, endothelial cells, immune cells, and hematopoietic progenitors actively participate in the formation of the bone metastatic niche [11,12]. The reciprocal interaction between disseminated tumor cells and the bone microenvironment gives rise to a pathological “vicious cycle,” in which tumor-derived factors stimulate osteoclastogenesis and/or aberrant osteoblastic activity, promoting bone remodeling, matrix degradation, and the release of molecules that further favor tumor progression [3,13].

It is now recognized that the extracellular matrix is not only a structural support but also a dynamic signaling platform that regulates tumor progression, mechanotransduction, angiogenesis, immunomodulation, and metastatic dissemination [14,15]. Therefore, proteoglycans (PGs) have emerged as critical regulators of communication between tumor cells and the stroma, as well as of the establishment and progression of BM [16,17]. PGs are structurally diverse glycoconjugates composed of a core protein covalently linked to one or more glycosaminoglycan (GAG) chains, including heparan sulfate, chondroitin sulfate, dermatan sulfate, keratan sulfate, and hyaluronan (HA)-associated complexes [17,18]. Heparan sulfate proteoglycans (HSPGs) and heparanase (HPSE) are particularly relevant in this context because they participate in extracellular matrix remodeling and regulate tumor–bone microenvironment interactions that facilitate metastatic progression [17]. Due to the high negative charge of their GAG chains and their interactive domains, PGs regulate the bioavailability and signaling of growth factors, cytokines, chemokines, integrins, and morphogens, modulating cellular processes such as adhesion, migration, proliferation, differentiation, angiogenesis, and the immune response [14,17].

Beyond their physiological functions in tissue organization and skeletal homeostasis, PGs are now recognized as active participants in tumor progression and metastasis [19]. Various PGs, including syndecans, glypicans, perlecan, versican, decorin, biglycan, members of the SPOCK family, and HA-dependent signaling networks, have been implicated in tumor invasion, metastatic niche formation, osteoclast activation, osteoblastic dysfunction, angiogenesis, tumor latency, and therapeutic resistance [11,16,19]. Furthermore, alterations in proteoglycan expression, ectodomain shedding, sulfation patterns, and enzymatic remodeling mediated by matrix metalloproteinases and HPSE profoundly modify the metastatic microenvironment and favor metastatic competence [17,20,21].

In BM, some proteoglycans have been shown to have specific mechanistic functions in tumor-bone interactions [3,22]. Versican G3 domain promotes migration, invasion, and epidermal growth factor receptor/extracellular signal-regulated kinase (EGFR/ERK)-mediated signaling in breast cancer cells, as well as altering osteoblastic differentiation [19,23]. Decorin has demonstrated anti-metastatic effects in preclinical models of breast and PCa by inhibiting tyrosine kinase receptor pathways, angiogenesis, tumor migration, and osteoclastic activity [19,24,25]. Perlecan/HSPG2, for its part, contributes to the metastatic progression of prostate cancer by regulating growth factor availability, stromal invasion, focal adhesion kinase (FAK) signaling, and desmoplastic remodeling of the bone niche [26,27,28]. Similarly, syndecan-1, syndecan-4, beta-glycan/TGFβ-RIII, and the HA-CD44 and HA/CD168-dependent pathways have been associated with metastatic colonization, tumor latency, therapeutic resistance, and bone progression of cancer [16,19,29].

Despite growing experimental evidence, the mechanistic integration of proteoglycan-mediated signaling pathways in bone metastasis remains incomplete, and their translational exploitation as biomarkers or therapeutic targets is still limited. Therefore, a comprehensive and up-to-date synthesis of proteoglycan-dependent mechanisms in the BM niche is needed.

In this review, we discuss proteoglycans as molecular regulators of BM, with particular emphasis on breast and PCa. We examine their roles in extracellular matrix remodeling, tumor–bone crosstalk, osteolytic and osteoblastic niche formation, angiogenesis, immune regulation, metastatic dormancy, therapy resistance, and emerging therapeutic opportunities. We also analyze how proteoglycan localization, glycosaminoglycan composition, sulfation patterns, ectodomain shedding, and proteolytic processing shape their context-dependent functions in metastatic bone disease.

## 2. Methods: Literature Search and Evidence Selection

### 2.1. Review Design

This article presents a narrative review that provides a mechanistic and translational synthesis of proteoglycan- and glycosaminoglycan-regulated processes in the BM. It was not designed as a systematic review or meta-analysis. Therefore, no PRISMA flow diagram, formal risk-of-bias assessment, or quantitative synthesis of evidence was performed. The objective was to integrate relevant experimental, translational, and clinical evidence for extracellular matrix remodeling, tumor-bone interaction, latency, immune regulation, treatment resistance, and therapeutic opportunities in skeletal metastatic disease.

### 2.2. Literature Search Strategy

For reproducibility, all searches were last performed on 31 May 2026. PubMed searches were performed using the representative strings listed in this section. Google Scholar was searched on the same date using relevance-based sorting and was used as a complementary discovery tool. Only English-language publications were considered. Because Google Scholar results are dynamic, the search was not used to produce reproducible quantitative counts of records. Searches combined proteoglycan- and glycosaminoglycan-related terms with bone metastasis- and tumor microenvironment-related terms. Representative search strings included: “proteoglycans AND bone metastasis”; “glycosaminoglycans AND bone metastasis”; “heparan sulfate proteoglycans AND bone metastasis”; “heparanase AND bone metastasis”; “hyaluronan OR CD44 OR RHAMM AND bone metastasis”; “perlecan OR HSPG2 AND prostate cancer bone metastasis”; “versican AND breast cancer bone metastasis”; “decorin AND bone metastasis”; “syndecan AND skeletal metastasis”; “SULF1 OR SULF2 AND cancer metastasis”; “proteoglycans AND dormancy AND bone metastasis”; and “extracellular matrix remodeling AND therapy resistance AND bone metastasis.” For Google Scholar, searches were used to identify highly cited, recent, and cross-disciplinary articles not readily captured through PubMed indexing. Because Google Scholar rankings are dynamic and vary by date, location, and indexing updates, Google Scholar was used as a complementary discovery tool rather than as a source for reproducible quantitative record counts.

### 2.3. Eligibility Criteria

We considered original research articles, reviews, and translational studies published in English that addressed at least one of the following topics: proteoglycans, glycosaminoglycans, heparan sulfate proteoglycans, hyaluronan-associated signaling, extracellular matrix remodeling, tumor–bone crosstalk, osteolytic or osteoblastic bone metastasis, metastatic dormancy or reactivation, immune regulation, therapy resistance, biomarkers, or therapeutic targeting. Particular emphasis was placed on studies involving breast cancer, prostate cancer, and other bone-tropic solid tumors. Multiple myeloma and primary bone tumors were considered only as comparative bone-niche contexts when they provided mechanistic information relevant to proteoglycan biology. Studies were excluded when they did not address proteoglycans, glycosaminoglycans, extracellular matrix remodeling, or bone metastatic biology; when they focused exclusively on non-malignant skeletal disease without relevance to metastasis; or when the mechanistic link to the review topic was too indirect.

### 2.4. Duplicate Handling and Evidence Selection

Because this was a narrative review, no formal risk-of-bias scoring was performed. Duplicate records retrieved from PubMed, Google Scholar, and reference lists were manually removed. Articles were prioritized based on their relevance to proteoglycan-dependent mechanisms in bone metastasis, with preference given to studies that provide direct evidence from BM models, patient-derived BM samples, bone-niche experimental systems, or mechanistic analyses clearly related to skeletal metastatic progression. Evidence was classified as direct bone-metastasis evidence, bone-niche experimental evidence, or indirect evidence extrapolated from primary tumors, non-bone metastatic models, or general cancer biology. In the revised manuscript, we distinguish direct evidence of BM from broader mechanistic evidence whenever possible.

### 2.5. Limitations of the Search Approach

Given the narrative nature of this review, the literature search was not intended to be exhaustive as in a systematic review. Exact numbers of records retrieved, screened, excluded, and included are therefore not reported.

## 3. Proteoglycans and Glycosaminoglycans: Functional Overview for Cancer Biology

PGs are large glycoconjugates, typically deposited in the cellular glycocalyx and extracellular matrix, where they serve as dynamic platforms that integrate biochemical, mechanical, and immunological signals within the tumor microenvironment [14,17]. They are currently recognized as active regulators of proliferation, adhesion, cell plasticity, migration, angiogenesis, immunomodulation, and metastatic dissemination [17]. The structural diversity of their protein domains, along with their glycosylation and sulfation patterns, enables them to modulate multiple signaling pathways associated with tumor progression and metastasis (Table 1 and Table 2) [30].

GAG chains are the main functional determinants of these molecules. Changes in the length, sulfation, and spatial organization of GAGs modify their affinity for ligands and alter the local availability of soluble mediators such as vascular endothelial growth factor (VEGF), transforming growth factor beta (TGF-β), fibroblast growth factor (FGF), hepatocyte growth factor (HGF), and chemokine (CXC Motif) ligand 12 (CXCL12), favoring invasion, angiogenesis, and remodeling of the metastatic niche [17,32,33]. In particular, enzymatic remodeling mediated by HPSE, sulfatases (SULF1/SULF2), and matrix metalloproteinases (MMPs) contributes to the release of pro-tumor factors and the reorganization of the extracellular matrix, promoting metastatic competence and therapeutic resistance [17,20,34,35].

Based on their subcellular location and structural organization, proteoglycans can be grouped into intracellular, cell-surface, pericellular, and extracellular proteoglycans [19,36,37]. Although this classification has structural relevance, understanding its biological functions within the tumor microenvironment is more important in cancer. Cell-surface proteoglycans, particularly syndecans and glypicans, participate in oncogenic signaling, cell adhesion, and tumor-stroma communication [16,19]. Alterations in syndecan-1 and -3 shedding expression have been linked to cell migration, epithelial–mesenchymal transition, drug resistance, and metastatic progression [38,39].

Pericellular proteoglycans, such as perlecan/HSPG2 and collagen XVIII, are critical components of basement membranes and specialized tissue niches [40]. These molecules regulate matrix organization, mechanotransduction, angiogenic signaling, and growth factor availability, playing key roles in tumor invasion and metastatic niche formation. Furthermore, products derived from their proteolytic processing, such as endostatin, possess antiangiogenic properties and modulate the tumor microenvironment.

Among the extracellular proteoglycans, versican, decorin, biglycan, lumican, and members of the SPOCK/testican family stand out, actively participating in extracellular matrix remodeling, collagen organization, tumor inflammation, and cell plasticity [41,42]. Versican promotes cell migration, invasion, and EGFR- and EMT-associated signaling, while decorin exerts antitumor effects by inhibiting tyrosine kinase receptors and downregulating angiogenesis and cell proliferation [43,44]. Furthermore, HA and its receptors CD44 and RHAMM/CD168 participate in adhesion, migration, tumor latency, and the formation of premetastatic niches [31,42,45]. Their involvement in oncogenic signaling, extracellular matrix organization, immune regulation, and tumor-stroma communication makes them key molecules for understanding metastatic progression and potential biomarkers and therapeutic targets in cancer, particularly in bone metastasis.

## 4. Bone Metastatic Niche: Proteoglycan-Relevant Cellular and Matrix Interfaces

Bone metastasis develops within a highly specialized microenvironment in which disseminated tumor cells interact with bone-resident cells, stromal components, immune populations, and extracellular matrix molecules [11,46]. In this review, the BM niche is considered specifically as a set of cellular and matrix interfaces that regulate proteoglycan- and GAG-dependent signaling (Table 3). Unlike many other metastatic organs, bone combines continuous remodeling, abundant vascularization, and a mineralized ECM enriched in matrix-stored growth factors, chemokines, adhesive ligands, and proteoglycans, creating conditions that support tumor-cell adhesion, survival, dormancy, and metastatic expansion [3,11,47,48].

Within this niche, osteoblasts, osteoclasts, osteocytes, stromal fibroblasts, endothelial cells, and immune cells are relevant not only as regulators of bone turnover, but also as producers, organizers, or remodelers of PG/GAG-rich matrices. Osteoblast-lineage and stromal cells contribute to endosteal niche architecture, osteogenic signaling, Receptor Activator of Nuclear Factor-κB Ligand/Osteoprotegerin (RANKL/OPG) balance, CXCL12 gradients, and ECM deposition, thereby influencing tumor-cell retention and latency [49,50,51,52]. Osteoclasts, by contrast, remodel the mineralized matrix and release growth factors such as TGF-β, insulin-like growth factors (IGFs), and BMPs, which can amplify tumor growth and feed the tumor–bone vicious cycle [53,54]. Osteocytes and mechanically responsive stromal cells further contribute to matrix organization and mechanotransduction, processes that are relevant because proteoglycans, HA-rich matrices, collagen-associated SLRPs, and basement-membrane HSPGs can regulate tissue stiffness, ligand availability, and cell–matrix signaling [30,49,54,55].

Disseminated tumor cells exploit these physiological bone-remodeling interfaces to establish a permissive metastatic niche [50,51,52]. Chemotactic and adhesive mediators such as CXCL12, osteopontin, vascular cell adhesion molecule 1 (VCAM-1), integrins, Jagged-1, TGF-β, BMPs, and receptor activator of nuclear factor-κB ligand (RANKL) regulate homing, adhesion, survival, metabolic adaptation, and osteoclast or osteoblast activation [52,56,57,58]. PG/GAG-dependent mechanisms intersect with many of these axes because heparan sulfate chains, chondroitin/dermatan sulfate proteoglycans, and HA-associated matrices can sequester, present, or release growth factors, morphogens, cytokines, and chemokines. Thus, proteoglycans should be interpreted as signaling organizers of the tumor–bone interface rather than as passive structural components of the ECM [17,30,42,59].

In osteolytic metastases, commonly observed in breast cancer and multiple myeloma, tumor-derived factors such as parathyroid hormone-related protein (PTHrP), IL-11, and TNF-α, which induce RANK-associated signaling, stimulate osteoclastogenesis and bone resorption [53,54]. This process releases matrix-stored TGF-β, insulin-like growth factors (IGFs), bone morphogenic proteins (BMPs), and other bioactive molecules that further support tumor proliferation and osteolytic progression [22,53,54]. PG/GAG remodeling contributes to this cycle through HPSE-mediated cleavage of heparan sulfate chains, MMP- and cathepsin-dependent proteolysis of ECM components, and HA-CD44/RHAMM-associated signaling, all of which may increase ligand availability, invasion, and adaptation to the bone niche [17,22,42,55]. In osteoblastic or mixed lesions, particularly in prostate cancer, proteoglycan remodeling is instead linked to aberrant matrix deposition, desmoplastic remodeling, osteogenic signaling, and mechanically altered osteoid [3,60]. Osteolytic and osteoblastic activities frequently coexist; therefore, the lesion phenotype should be interpreted as a dynamic balance among resorption, abnormal bone formation, and matrix remodeling rather than as a binary state [3,60].

Immune cells also shape the PG/GAG-dependent bone metastatic niche. Macrophages, T cells, NK cells, dendritic cells, myeloid-derived suppressor cells (MDSCs), and neutrophils can either restrict metastatic growth or promote immunosuppression, angiogenesis, osteoclastogenesis, and ECM remodeling, depending on their activation state [61]. Inflammatory mediators such as IL-6, TNF-α, CXCLs, and TGF-β promote osteoclast activation and tumor progression, while also regulating matrix-remodeling enzymes and proteoglycan-associated signaling [47,62]. In prostate cancer bone metastasis models, M2-like macrophages have been linked to desmoplastic remodeling and to stromal regulation of perlecan/HSPG2 and sulfatases, connecting immune polarization with heparan sulfate remodeling and tumor-supportive ECM organization [27,39]. Therefore, the immune niche should be discussed in this review primarily in terms of its effects on proteoglycan expression, GAG editing, proteolysis, chemokine presentation, and matrix stiffness.

**Table 3:** Immune–proteoglycan/GAG contributions to the skeletal metastatic niche.

Immune-Cell Population or Axis	Main Mediators or Mechanisms	Proteoglycan/GAG–ECM Interface	Dominant Bone-Metastasis Output	Ref.
Macrophage lineage: TAMs, MAMs, osteomacs, and efferocytic macrophages	CCL2/CCR2, CSF-1/CSF-1R, IL-6, IL-10, TGF-β, EGF, PDGF, VEGF, MMPs, cathepsins, ARG1, PGE2, PD-L1; efferocytosis-associated NF-κB/STAT3 activation.	Remodel PG/GAG-rich ECM through proteases and cytokines. M2-like macrophage–fibroblast interactions can regulate perlecan/HSPG2, SULF1/2, HA-rich matrices, and desmoplastic remodeling.	Tumor-cell survival, invasion, angiogenesis, osteoclastogenesis, immune evasion, and metastatic outgrowth.	[13,27,39,61,63,64]
MDSCs	ARG1, iNOS/NO, ROS, IL-10, TGF-β, CXCR4 signaling, amino-acid depletion, Treg expansion; in some models, differentiation into osteoclast-like cells.	Promote inflammatory and hypoxic matrix remodeling and may amplify osteoclast-dependent release of matrix-stored growth factors.	T-cell and NK-cell suppression, osteolysis, angiogenesis, and immune escape.	[13,39,61,64,65]
Neutrophils and TANs	CXCLs, CXCR4, VEGF, MMP9, elastase, ROS, and NETs in selected contexts.	Support proteolytic ECM remodeling, vascular permeability, tumor-cell adhesion, and early niche conditioning.	Premetastatic niche formation, extravasation, colonization, and context-dependent pro- or antitumor activity.	[13,61,63,66,67]
Effector lymphocytes: CD8+ T cells, Th1 cells, NK cells, and NKT cells	Perforin, granzymes, Fas/FasL, IFN-γ, NKG2D, DNAM-1; inhibited by TGF-β, PD-1/PD-L1, ROS, MDSCs, and chronic antigen exposure.	Bone resorption releases matrix-stored TGF-β, which can suppress cytotoxic function. Stiff or HA-rich matrices may further limit immune-cell infiltration and activity.	Antitumor surveillance, restriction of early seeding, reduced tumor burden, and delayed metastatic expansion when functional.	[13,61,63,65,68,69]
Regulatory and tolerogenic adaptive axis: Tregs, tolerogenic DCs, and Th2-biased responses	FOXP3, CTLA-4, IL-10, TGF-β, impaired MHC-I/MHC-II antigen presentation, IL-4, IL-13.	Immunosuppressive cytokines reshape stromal remodeling, macrophage polarization, and PG/GAG-dependent inflammatory signaling.	Tumor persistence, immune escape, reduced T-cell priming, and context-dependent effects on osteoclastogenesis.	[13,61,63]
IL-17-associated osteoimmune axis: Th17 and Tc17 cells	IL-17, TNF-α, RANKL, and induction of stromal/osteoblastic RANKL expression.	Drives inflammatory osteoclastogenesis and matrix turnover, favoring release of TGF-β, IGFs, BMPs, and other matrix-stored factors.	Osteolytic progression, growth-factor release, and metastatic colonization.	[13,61,63,66]
Immune–stromal PG/GAG remodeling axis: TAMs–CAFs–ECM	Perlecan/HSPG2, SULF1/2, HPSE, WNT3A, MMPs, ADAMTS proteases, cathepsins, LOX/LOXL2, cytokine-induced remodeling.	Directly links immune polarization with heparan sulfate editing, proteoglycan cleavage, HA/versican-rich inflammation, collagen remodeling, and matrix stiffness.	Desmoplasia, immune exclusion, dormancy/reactivation, therapy resistance, and niche stratification.	[13,27,61,64,65,70,71,72,73,74,75,76]

Abbreviations: ADAMTS, a disintegrin and metalloproteinase with thrombospondin motifs; ARG1, arginase 1; BMPs, bone morphogenetic proteins; CAFs, cancer-associated fibroblasts; CCL, C-C motif chemokine ligand; CCR, C-C motif chemokine receptor; CSF-1, colony-stimulating factor 1; CSF-1R, colony-stimulating factor 1 receptor; CTLA-4, cytotoxic T-lymphocyte-associated protein 4; CXCL, C-X-C motif chemokine ligand; CXCR, C-X-C motif chemokine receptor; CD8+, cluster of differentiation 8-positive T cells; DCs, dendritic cells; DNAM-1, DNAX accessory molecule 1; ECM, extracellular matrix; EGF, epidermal growth factor; Fas, Fas cell surface death receptor; FasL, Fas ligand; FOXP3, forkhead box P3; GAG, glycosaminoglycan; HA, hyaluronan; HPSE, heparanase; HSPG2, heparan sulfate proteoglycan 2/perlecan; IFN-γ, interferon gamma; IGFs, insulin-like growth factors; IL, interleukin; iNOS, inducible nitric oxide synthase; LOX/LOXL2, lysyl oxidases/lysyl oxidase-like 2; MAMs, metastasis-associated macrophages; MDSCs, myeloid-derived suppressor cells; MHC, major histocompatibility complex; MMPs, matrix metalloproteinases; NETs, neutrophil extracellular traps; NF-κB, nuclear factor kappa B; NK cells, natural killer cells; NKG2D, natural killer group 2 member D; NKT cells, natural killer T cells; NO, nitric oxide; PD-1, programmed cell death protein 1; PDGF, platelet-derived growth factor; PD-L1, programmed death-ligand 1; PG, proteoglycan; PGE2, prostaglandin E2; RANKL, receptor activator of nuclear factor-κB ligand; ROS, reactive oxygen species; STAT3, signal transducer and activator of transcription 3; SULF1/2, sulfatase 1/2; TAMs, tumor-associated macrophages; TANs, tumor-associated neutrophils; Tc17, IL-17-producing cytotoxic T cells; TGF-β, transforming growth factor beta; Th, T helper cell; Tregs, regulatory T cells; VEGF, vascular endothelial growth factor; WNT3A, Wingless-type MMTV integration site family member 3A.

Several proteoglycan-centered examples illustrate how these interfaces operate. Perlecan/HSPG2 can function as a basement membrane and pericellular reservoir for growth factors and has been implicated in stromal invasion and desmoplastic remodeling in prostate cancer bone metastases [27,28,76,77,78]. Versican, particularly through its G3 domain, promotes breast cancer cell migration, EGFR-mediated signaling, and altered osteoblast behavior, supporting its relevance in breast cancer adaptation to bone [23,26]. Decorin, in contrast, has shown anti-metastatic activity in preclinical breast and prostate cancer models through antagonism of receptor tyrosine kinase signaling, inhibition of angiogenesis, and modulation of osteoclast-associated remodeling [59,76,79]. HA is not a proteoglycan, but a free, non-sulfated extracellular/pericellular GAG that functionally cooperates with hyalectans and receptors such as CD44 and RHAMM to regulate adhesion, motility, stemness, immune modulation, and therapy resistance [31,42].

The bone metastatic niche also influences tumor dormancy and late recurrence. Disseminated tumor cells may remain quiescent for prolonged periods under the influence of osteoblast-derived factors, ECM organization, TGF-β family members, and endosteal niche signals [80,81,82]. In prostate cancer, osteoblast-derived GDF10 and TGFβ2 can induce tumor-cell dormancy through the TGFβ-RIII/betaglycan–p38MAPK–RB pathway [83,84]. However, the broader causal role of proteoglycan remodeling in dormancy maintenance or escape remains less well defined. Changes in GAG sulfation, HPSE activity, HA-CD44/RHAMM signaling, proteoglycan fragmentation, matrix stiffness, inflammatory remodeling, or osteoclast-dependent matrix turnover may contribute to the transition from quiescence to metastatic outgrowth, but these mechanisms require further validation across tumor types [59,80,82,85,86].

Taken together, the bone metastatic niche is best understood as a dynamic tumor–bone–immune–matrix interface, in which proteoglycans and GAG-associated pathways regulate ligand availability, adhesion, mechanotransduction, inflammatory remodeling, dormancy, reactivation, and therapeutic vulnerability [42,61]. Proteoglycan-dependent dormancy and reactivation are discussed in detail in Section 5.6.

## 5. Proteoglycans as Regulators of the Metastatic Cascade

**Figure 1:**
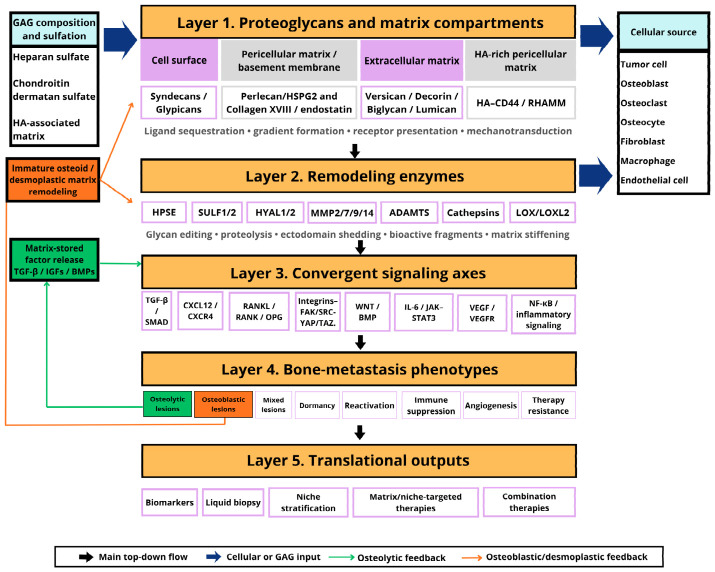
Proteoglycan remodeling as a convergent regulatory network in bone metastasis. Abbreviations: ADAMTS, a disintegrin and metalloproteinase with thrombospondin motifs; BMP, bone morphogenetic protein; CD44, cluster of differentiation 44; CXCL12, C-X-C motif chemokine ligand 12; CXCR4, C-X-C motif chemokine receptor 4; ECM, extracellular matrix; FAK, focal adhesion kinase; GAG, glycosaminoglycan; HA, hyaluronan; HPSE, heparanase; HSPG2, heparan sulfate proteoglycan 2; HYAL, hyaluronidase; IGF, insulin-like growth factor; IL-6, interleukin-6; JAK, Janus kinase; LOX/LOXL2, lysyl oxidase/lysyl oxidase-like 2; MMP, matrix metalloproteinase; NF-κB, nuclear factor kappa B; OPG, osteoprotegerin; RANK, receptor activator of nuclear factor-κB; RANKL, receptor activator of nuclear factor-κB ligand; RHAMM, receptor for hyaluronan-mediated motility; SMAD, suppressor of mothers against decapentaplegic proteins; Src, proto-oncogene tyrosine-protein kinase Src; STAT3, signal transducer and activator of transcription 3; SULF1/2, sulfatase 1/2; TGF-β, transforming growth factor beta; VEGF, vascular endothelial growth factor; VEGFR, vascular endothelial growth factor receptor; WNT, wingless/integrated signaling pathway.

Proteoglycans should be interpreted as regulatory nodes in the metastatic cascade rather than as passive structural components of the extracellular matrix [42]. In bone metastasis, their functions depend on core protein identity, glycosaminoglycan composition, sulfation patterns, spatial localization, enzymatic processing, cellular source, and inflammatory or mechanical context [5,37,51,75,87]. This is particularly relevant in the skeletal niche, where tumor cells interact with osteoblasts, osteoclasts, osteocytes, endothelial cells, fibroblasts, macrophages, lymphocytes, hematopoietic progenitors, and a mineralized extracellular matrix enriched in growth factors and chemokines [46,87,88,89]. A mechanistic interpretation of proteoglycan biology therefore requires moving beyond a molecule-by-molecule description. In this section, we propose an integrative framework in which proteoglycan remodeling coordinates tumor–bone communication through five interconnected regulatory layers: localization, glycosaminoglycan composition and sulfation, enzymatic processing, cellular source, and convergence on common metastatic signaling axes (Figure 1). This framework helps explain how structurally diverse proteoglycans can regulate osteolytic and osteoblastic niche formation, metastatic dormancy, reactivation, immune escape, angiogenesis, and therapeutic resistance.

### 5.1. An Integrative Proteoglycan-Centered Model of Bone Metastasis

Rather than acting as isolated matrix components, proteoglycans should be interpreted as an interconnected signaling network that links tumor cells, bone-resident cells, immune populations, vascular cells, and the extracellular matrix [37,90,91,92]. Heparan sulfate proteoglycans such as syndecans, glypicans, and perlecan/HSPG2 regulate ligand sequestration, gradient formation, and receptor presentation [93,94,95,96], whereas hyaluronan–versican–CD44/RHAMM complexes shape pericellular matrix organization, motility, stemness-associated programs, and inflammatory signaling [31,76,97,98,99]. Small leucine-rich proteoglycans, including decorin, biglycan, and lumican, further modulate collagen architecture, receptor tyrosine kinase signaling, innate immune activation, matrix stiffness, and osteoclast-associated remodeling [5,18,41,44,74,75,100,101]. Enzymatic editing by heparanase, sulfatases, hyaluronidases, MMPs, ADAMTS proteases, cathepsins, and LOX/LOXL enzymes converts this matrix network into a dynamic regulator of osteolytic and osteoblastic progression, metastatic dormancy, immune escape, and therapy resistance [89,101,102,103,104,105,106]. Therefore, this network can be organized according to five mechanistic layers, as shown in Figure 1. First, localization determines the type of signaling interface [31,37,91,93,95,96,107,108]. Cell-surface proteoglycans, such as syndecans and glypicans, act as co-receptors that regulate adhesion, growth factor signaling, integrin activation, and tumor–stroma communication [93,94,96,108,109]. Pericellular and basement-membrane proteoglycans, particularly perlecan/HSPG2 and collagen XVIII, regulate compartmentalized ligand storage, angiogenesis, and invasion across matrix barriers [95,107]. Extracellular proteoglycans, including versican, decorin, biglycan, and lumican, regulate collagen organization, matrix hydration, inflammation, and mechanotransduction [41,44,74,75,76,100,110]. Hyaluronan is not a proteoglycan in the strict structural sense, but it is functionally integrated into the proteoglycan network through hyalectans and receptors such as CD44 and RHAMM [31,76,98,99,110].

Second, glycosaminoglycan composition and sulfation act as biochemical codes that determine ligand binding and signaling intensity [92,93,94,109,111]. Heparan sulfate chains regulate the availability of FGF, VEGF, HGF, WNT, BMPs, TGF-β-related ligands, and CXCL12 [93,94,103,109], whereas chondroitin sulfate and dermatan sulfate proteoglycans regulate matrix organization, inflammatory signaling, and collagen-associated functions [41,44,74,75,76,100,110,111]. Changes in glycosaminoglycan length, sulfation pattern, and spatial organization can therefore convert the extracellular matrix from a structural scaffold into a reservoir, filter, or amplifier of metastatic signaling [92,93,94,102,103,109,111,112].

Third, enzymatic processing dynamically rewires the proteoglycan network. Heparanase releases heparan sulfate-bound growth factors and modifies matrix barriers; SULF1 and SULF2 edit 6-O-sulfation and alter ligand availability [113]; hyaluronidases remodel hyaluronan-rich pericellular matrices; MMPs, ADAMTS proteases, and cathepsins cleave core proteins, ectodomains, or associated matrix components; and LOX/LOXL enzymes increase collagen crosslinking and matrix stiffness [102,103,104,105,112,114,115,116,117]. These processes generate bioactive fragments, expose cryptic binding sites, and modulate mechanotransduction, thereby influencing invasion, osteoclastogenesis, aberrant osteoblast activation, angiogenesis, and immune cell recruitment [112,116,117,118,119].

Fourth, the cellular source of proteoglycans determines their local function [5,47,89,90,101,106,112]. Tumor-derived proteoglycans may promote invasion, survival, epithelial–mesenchymal plasticity, and resistance to therapy [37,90,97,109,112,120]. Osteoblast- and stromal-derived proteoglycans can regulate dormancy, osteogenic signaling, and tumor-cell adhesion within the endosteal niche [5,89,101,106,119,121]. Osteoclast-associated remodeling releases matrix-stored factors that amplify tumor growth [5,89,101,106,121]. Fibroblasts and cancer-associated fibroblasts contribute to desmoplastic matrix deposition, whereas macrophages and other immune cells regulate proteoglycan remodeling through inflammatory cytokines, proteases, heparanase, and sulfatases [5,74,76,102,103,106,110,112,115]. Endothelial cell-associated proteoglycans further influence vascular permeability, angiogenesis, and tumor cell extravasation [5,89,90,93,95,101,106,107].

Fifth, these layers converge on a limited number of metastatic signaling axes. Proteoglycan remodeling modulates TGF-β/SMAD, CXCL12/CXCR4, RANKL/RANK/OPG, integrin–FAK/Src, WNT/BMP, IL-6/JAK–STAT3, VEGF/VEGFR, and NF-κB-dependent inflammatory signaling [5,31,74,76,89,93,94,98,101,102,103,106]. These pathways do not operate independently; instead, they cooperate to determine whether disseminated tumor cells remain dormant, reactivate, induce osteolytic destruction, stimulate aberrant osteoblastic bone formation, evade immune surveillance, or acquire therapeutic resistance [5,97,102,106,112,119,120].

### 5.2. Proteoglycan Remodeling in Osteolytic Versus Osteoblastic Lesions

Proteoglycan remodeling provides a mechanistic framework to distinguish osteolytic, osteoblastic, and mixed bone metastases. Although these phenotypes are often associated with specific tumor types, they should not be interpreted as mutually exclusive biological states. Osteolytic lesions, frequently observed in breast cancer and multiple myeloma, are dominated by osteoclast activation, bone resorption, and release of matrix-stored growth factors [5,53,54,122,123,124]. Osteoblastic lesions are associated with advanced prostate cancer and characterized by aberrant bone formation, desmoplastic remodeling, and deposition of structurally abnormal osteoid [60,125,126]. Osteoclastic and osteoblastic activities frequently coexist, indicating that lesion phenotype reflects the relative balance between resorption, bone formation, and matrix remodeling rather than a binary state [5,124,126]. In osteolytic metastases, HPSE, MMPs, hyaluronidases, cathepsins, HA-CD44/RHAMM signaling, syndecans, versican, and perlecan/HSPG2 contribute to matrix degradation, proteoglycan cleavage, and release of growth factors stored in the mineralized bone matrix [17,22,31,42,94,95,102,104,114,116]. These processes increase the local availability of TGF-β, IGFs, BMPs, VEGF, FGF, HGF, and chemokines, thereby amplifying tumor-cell survival, proliferation, PTHrP-associated signaling, RANKL availability, osteoclastogenesis, and the tumor–bone vicious cycle [5,22,52,53,94,119,127,128,129,130].

In osteoblastic metastases, proteoglycan remodeling contributes to aberrant matrix deposition, osteogenic signaling, and desmoplastic niche formation [26,27,28,60,95,125,126]. Perlecan/HSPG2 can act as a basement membrane and pericellular reservoir for FGF, VEGF, HGF, WNTs, and BMP-related signals, whereas SULF1 and SULF2 modify heparan sulfate sulfation patterns and regulate the availability of WNT, FGF, VEGF, and other heparan sulfate-bound ligands [27,35,94,95,103,107,109,131,132,133]. Glypicans, syndecan-4, integrins–FAK/Src, and WNT/BMP signaling may further contribute to osteogenic programs, focal adhesion dynamics, and mechanotransduction [119,134,135,136]. LOX/LOXL2-mediated matrix crosslinking can increase stiffness and promote desmoplastic remodeling [15,105,117,137]. Despite these differences, osteolytic and osteoblastic lesions converge on shared proteoglycan-regulated axes, including TGF-β/SMAD, CXCL12/CXCR4, RANKL/RANK/OPG, integrin–FAK/Src, WNT/BMP, IL-6/JAK–STAT3, VEGF/VEGFR, and NF-κB-dependent inflammatory signaling [5,94,118,119,127,129,138,139,140,141]. Thus, proteoglycan remodeling does not merely accompany lesion formation; it helps coordinate the molecular balance between bone destruction, aberrant bone formation, dormancy, immune regulation, and therapeutic resistance [138,142,143,144].

### 5.3. Enzymatic Editing and Proteolytic Remodeling of the Proteoglycan Network

The functional state of the proteoglycan network is continuously reshaped by matrix-remodeling enzymes [102,103,119,145]. HPSE cleaves heparan sulfate chains and releases heparan sulfate-bound growth factors, chemokines, and morphogens [102,146]. SULF1 and SULF2 edit extracellular 6-O-sulfation of heparan sulfate, thereby changing ligand affinity and signaling gradients [103,145,147]. HYAL1 and HYAL2 remodel hyaluronan-rich pericellular matrices and modify CD44/RHAMM-dependent signaling [31,148]. MMP2, MMP-7, MMP9, MMP14, ADAMTS proteases, and cathepsins cleave proteoglycan core proteins, ectodomains, basement-membrane components, and associated extracellular matrix proteins [42,112,114,116,149]. LOX and LOXL2 cross-link collagen and elastin, thereby increasing matrix stiffness and mechanotransduction via integrin–FAK/Src and yes-associated protein/transcriptional coactivator with PDZ-binding motif (YAP/TAZ) signaling [105,150,151,152].

This enzymatic remodeling should be interpreted not only as extracellular matrix degradation but also as glycan editing and proteolytic signaling [102,103,114,115,145,146,147]. Protease-mediated cleavage can generate soluble ectodomains, expose cryptic binding sites, release matrix-bound ligands, and produce bioactive fragments with pro- or anti-metastatic functions [114,116,153,154,155]. In prostate cancer bone metastasis models, perlecan/HSPG2 is particularly relevant because HPSE and proteases can disrupt perlecan-rich matrices, release or expose bioactive cues, and MMP-7-mediated cleavage of perlecan or the perlecan–Sema3A complex shifts FAK-dependent cell behavior toward dyscohesion, invasion, and progression to lethal bone disease [26,28,123,156]. M2-like macrophages can also increase stromal SULF1 and HSPG2 expression in perlecan-modified triculture models, linking ECM remodeling, inflammation, and desmoplastic bone stroma [27].

### 5.4. Convergence on Common Metastatic Signaling Axes

Although individual proteoglycans differ in structure, localization, glycosaminoglycan composition, and cellular source [21,37,91,111], their functions converge on a limited set of signaling axes that control skeletal metastatic progression [5,42,101,106,119]. Their core proteins, sulfated glycosaminoglycan chains, soluble ectodomains, and proteolytic fragments regulate the sequestration, presentation, and release of signaling molecules such as FGF2/FGFR, VEGF-A/VEGFR, PDGF/PDGFR, HGF/MET, EGF/EGFR, IGF/IGF1R, TGF-β/BMP/SMAD, WNT/β-catenin, Sonic Hedgehog signaling/GLI family zinc finger transcription factors (SHH/GLI), IL-6/JAK–STAT3, TNF-α/NF-κB, CXCL12/CXCR4, CCL2/CCR2, CXCL8/IL-8, RANKL/RANK/OPG, PTHrP/GLI2, Jagged1/Notch, VCAM-1/α4β1, osteopontin–integrins/CD44, and integrins β1/β3–FAK–Src–PI3K–AKT [5,37,53,143,144,157,158]. In this way, proteoglycans integrate biochemical, mechanical, and immunological signals that regulate adhesion, migration, invasion, angiogenesis, immune evasion, latency, therapeutic resistance, and colonization of distant organs [17,157,159,160]. Table 4 summarizes how proteoglycan- and GAG-regulated axes converge on shared metastatic signaling pathways and biological outputs in the bone microenvironment.

This convergence is particularly relevant in bone metastasis because the bone niche combines a mineralized matrix rich in growth factors, an immunologically active bone marrow, and constant remodeling dependent on osteoblasts, osteoclasts, osteocytes, endothelial cells, fibroblasts, macrophages, neutrophils, lymphocytes, and hematopoietic progenitors [46,51,138]. Within this niche, molecules such as CXCL12, integrins, osteopontin, VCAM-1, TGF-β, Jagged1, and RANKL participate in the adhesion, chemotaxis, survival, and expansion of disseminated tumor cells [139,140,177,178]. Thus, proteoglycans such as versican/VCAN, syndecans/SDC1–4, glypicans/GPCs, perlecan/HSPG2, agrin, collagen XVIII/endostatin, serglycin, and neuron-glial antigen 2/chondroitin sulfate proteoglycan 4 (NG2/CSPG4), along with the HA-CD44/RHAMM axis, can promote a permissive matrix for tumor colonization [37,42,95,142].

Several examples illustrate this functional convergence. In breast cancer bone metastasis models, the G3 domain of versican has been associated with adhesion, migration, proliferation, tumor metastatic burden, and activation of EGFR/ERK, EGFR/JNK, and AKT [23,162]. The HA-CD44 axis can activate PI3K–AKT and promote growth, motility, and invasion. Furthermore, the HA-CD44–ZEB1–HAS2 circuit contributes to EMT and prometastatic niche formation, while RHAMM/CD168 has been linked to ROCK1, GRB2-associated-binding protein 1 (Gab-1), PI3K, phosphatidylinositol 3-kinase catalytic subunit alpha (p110α), and eukaryotic translation initiation factor 4E family member 3 (eIF4E3) signaling in prostate cancer progression to bone [45,163,164,165]. HA is not a proteoglycan; it is a free, non-sulfated extracellular or pericellular glycosaminoglycan functionally associated with hyalectans and receptors such as CD44 and RHAMM/CD168 [37,166,167].

In prostate cancer bone metastasis models, perlecan/HSPG2 is particularly relevant because HPSE and proteases can disrupt perlecan-rich matrices and release or expose bioactive cues; in addition, MMP-7-mediated cleavage of perlecan or the perlecan–Sema3A complex shifts FAK-dependent cell behavior toward dyscohesion, invasion, and progression to lethal bone disease [26,137,161]. M2-like macrophages can increase stromal SULF1 and HSPG2 expression in perlecan-modified triculture models, linking ECM remodeling, inflammation and desmoplastic bone stroma [27,113,191,192]. Thus, extracellular proteolysis functions not only as barrier degradation, but also as proteolytic signaling that promotes tumor growth, immune escape, dormancy reactivation, and therapy resistance [143].

### 5.5. Context-Dependent Functions: Metastatic Drivers, Suppressive Barriers, Dormancy, Immune Escape, and Therapy Resistance

Proteoglycans display highly context-dependent functions in cancer progression and bone metastasis [37,42,75,142,144,193]. Several proteoglycans and glycan-associated axes are commonly linked to prometastatic behavior. Versican, syndecans, perlecan/HSPG2, serglycin, and the HA-CD44/RHAMM axis are commonly associated with migration, invasion, angiogenesis, inflammation, colonization, and resistance to therapy [39,76,95,99,194]. These associations should be interpreted in a tissue- and stage-specific manner, because the same molecule may support tumor expansion in one microenvironment while limiting progression or maintaining dormancy in another. Conversely, decorin and some small leucine-rich proteoglycans (SLRPs) can exert suppressive effects by antagonizing tyrosine kinase receptors and negatively modulating pathways such as EGFR, ERBB2/HER2, MET, VEGFR2, WNT/β-catenin, HIF-1α/VEGFA, and TGF-β, although these effects depend on the tumor, tissue, metastatic stage, or even the state of matrix remodeling [37,44,59,79,169]. These suppressive effects are also context-sensitive and may be altered by tumor type, metastatic site, matrix composition, and enzymatic remodeling.

A major limitation in the field is that much of the mechanistic evidence derives from in vitro models, xenografts, engineered three-dimensional cultures, or simplified experimental systems [48,138,195,196,197]. Although these approaches have clarified how proteoglycans regulate tumor–matrix interactions, clinical validation of proteoglycans as bone-metastasis-specific biomarkers or therapeutic targets remains limited. At present, clinical biomarker frameworks for bone metastasis are still dominated by bone turnover markers, tumor lineage markers, imaging-based assessment, and liquid biopsy approaches, whereas proteoglycan signatures and HSPG-targeted strategies remain mostly investigational [95,142,159,161,185]. Future studies should move beyond single-molecule interpretations and integrate matrisome profiling, spatial proteomics, single-cell and spatial transcriptomics, glycomics/GAGomics, degradomics, and mineralized three-dimensional multicellular models [182,198,199,200,201,202,203,204]. These approaches are particularly important because the metastatic bone niche contains tumor cells, osteoblasts, osteoclasts, osteocytes, endothelial cells, macrophages, lymphocytes, hematopoietic progenitors, and a mineralized extracellular matrix. Such integrative models may help distinguish proteoglycans that actively drive skeletal metastasis from those that act as antitumor barriers or context-dependent regulators of dormancy, reactivation, immune escape, and therapeutic resistance.

From a therapeutic perspective, proteoglycans should therefore be viewed as regulatory components of tumor–bone–immune–matrix crosstalk rather than merely structural elements of the extracellular matrix [48,138]. Promising strategies include targeting enzymatic matrix remodeling, glycosaminoglycan editing, HA synthesis and degradation, proteoglycan shedding, and downstream mechanotransduction pathways that sustain metastatic adaptation [35,84,113,127,141]. In prostate cancer models, recent proteomic and functional studies have linked perlecan/HSPG2 to matrisome remodeling, integrin-associated adhesion signaling and radioresistance, supporting the view that proteoglycans should be studied as functional components of therapeutic resistance rather than merely structural markers [5,116,143]. Taken together, this context-dependent perspective positions proteoglycans as dynamic regulators of metastatic plasticity, rather than fixed drivers or suppressors, and supports their inclusion in mechanistic and translational models of bone metastasis [183]. The following sections apply this integrative framework to specific tumor contexts, beginning with breast cancer, where proteoglycan remodeling is closely linked to osteolytic progression, HA-CD44/RHAMM signaling, versican-rich matrix remodeling, osteoclast activation, and the tumor–bone vicious cycle.

### 5.6. Proteoglycan-Dependent Dormancy and Reactivation

Metastatic dormancy in bone is not a passive state but a dynamic equilibrium between disseminated tumor cells and the endosteal, stromal, immune, vascular, and extracellular matrix compartments of the bone metastatic niche [80,81,82,101,121]. Proteoglycans and GAG-associated matrices should be interpreted as regulators of dormancy induction, dormancy maintenance, and reactivation, because they modulate ligand availability, receptor engagement, adhesion, matrix stiffness, inflammatory signaling, and proteolytic remodeling. However, the strength of the evidence differs across mechanisms and tumor types. The most direct bone-niche evidence currently supports a role for TGFβ-RIII/betaglycan-dependent signaling in prostate cancer dormancy, whereas the causal contribution of broader changes in proteoglycan expression, GAG sulfation, ectodomain shedding, heparanase activity, and proteoglycan fragmentation remains less well validated and should be interpreted with caution [80,81,82,83,84,85,86,179,180,181].

Dormancy induction occurs when disseminated tumor cells enter a quiescent or slow-cycling state after seeding the bone marrow. Osteoblast-lineage cells and endosteal stromal cells are central to this process because they provide adhesive, paracrine, and matrix-associated signals that restrain proliferation and favor long-term survival [80,81,82,121,179]. Among proteoglycan-related mechanisms, the TGFβ-RIII/betaglycan axis provides the clearest example of direct bone-niche dormancy regulation. In prostate cancer models, osteoblast-derived TGFβ2 and GDF10 activate TGFβ-RIII/betaglycan in tumor cells, leading to p38MAPK activation, RB phosphorylation, cell-cycle arrest, and a dormant phenotype [83,84,179,180,181]. This mechanism links an osteoblast-derived dormancy signal to a cell-surface proteoglycan co-receptor and provides strong evidence that proteoglycan-associated signaling can actively induce tumor-cell quiescence in the bone niche. Other dormancy-promoting cues, including BMP-related signaling, CXCL12/CXCR4 retention, integrin-mediated adhesion, osteopontin/CD44 interactions, and endosteal matrix organization, may also intersect with proteoglycan and GAG-dependent ligand presentation, although the causal role of individual proteoglycans in these axes is less direct [50,51,56,80,81,82,121,177].

Maintenance of dormancy requires sustained survival without overt metastatic expansion; proteoglycans may contribute to this state by stabilizing growth-factor gradients, maintaining pericellular and basement-membrane architecture, and regulating the balance between pro-quiescent and pro-mitogenic signaling. Heparan sulfate proteoglycans can sequester and present ligands such as FGFs, VEGF, HGF, WNTs, BMPs, TGF-β family members, and CXCL12, thereby shaping local signaling thresholds within the endosteal and marrow niches [93,94,95,101,109,121]. Perlecan/HSPG2 may be particularly relevant because its basement membrane and pericellular localization allow it to regulate growth factor storage, matrix boundaries, invasion barriers, and stromal organization [95,131,132,133]. HA-associated matrices may also influence dormancy-related phenotypes through CD44/RHAMM-dependent adhesion, survival, stemness, and motility programs [31,97,98,99,121]. SlRPs may further influence dormancy by modifying collagen organization, matrix stiffness, TGF-β availability, receptor tyrosine kinase signaling, and inflammatory tone [44,59,74,75,100]. Despite the above, current evidence supports a regulatory function of the bone niche, rather than constituting definitive proof that a specific proteoglycan is required to maintain latency in all tumor types.

Reactivation occurs when dormant tumor cells resume proliferation and progress toward clinically detectable bone metastasis; this transition is likely driven by coordinated changes in osteoclast activity, osteoblast function, inflammation, immune suppression, vascular remodeling, and extracellular matrix degradation [80,81,82,83,84,85,86,101,138]. Proteoglycan remodeling may contribute to reactivation by converting the matrix from a quiescence-supporting scaffold into a source of growth-promoting and invasion-promoting signals. Heparanase-mediated cleavage of heparan sulfate chains can release matrix-bound growth factors, chemokines, and morphogens, while SULF1/SULF2-dependent editing of heparan sulfate sulfation can alter WNT, FGF, VEGF, and other ligand gradients [34,35,102,103,113,145,146,147]. MMPs, ADAMTS proteases, cathepsins, and hyaluronidases can cleave proteoglycan core proteins, remodel HA-rich matrices, release soluble ectodomains, expose cryptic binding sites, and generate bioactive fragments or matrikines with pro- or anti-metastatic effects [104,112,114,115,116,153,154,155]. These processes can shift signaling toward ERK, FAK/Src, PI3K/AKT, WNT/BMP, IL-6/JAK–STAT3, NF-κB, and YAP/TAZ-dependent programs, thereby favoring proliferation, invasion, immune escape, angiogenesis, and resistance to therapy [75,101,119,143,183,184]. The reactivation process is also shaped by tumor type; in prostate cancer, dormancy and reactivation are closely linked to osteoblast-derived TGFβ2/GDF10–TGFβ-RIII/betaglycan–p38MAPK–RB signaling, perlecan/HSPG2-rich desmoplastic matrices, MMP-7–perlecan/Sema3A–FAK signaling, and SULF1/SULF2-dependent modulation of heparan sulfate-bound WNT3A in bone-niche models [27,28,83,84,103,156,161,179,180,181]. In breast cancer, osteoclast-mediated expansion of indolent micrometastases, VCAM-1/α4β1 interactions, HA-CD44/RHAMM signaling, versican-rich remodeling, and decorin-dependent suppression of skeletal metastasis [59,121,162,165,170,178]. In multiple myeloma, osteoclast-mediated remodeling of the endosteal niche has been shown to reactivate dormant tumor cells, supporting the broader concept that bone turnover and matrix remodeling can regulate escape from dormancy [86].

Proteoglycan-dependent dormancy should be considered a stage-specific and context-dependent process. Future studies should therefore combine gain- and loss-of-function approaches with mineralized three-dimensional bone-niche models, spatial proteoglycan/GAG mapping, degradomics, and patient-derived bone metastasis specimens to determine which proteoglycan alterations are merely associated with dormancy and which are causal drivers of late skeletal relapse [80,81,82,195,196,197,198,199,200,201,202,203,204].

## 6. Breast Cancer Bone Metastasis

Bone metastasis in breast cancer represents one of the most relevant scenarios for studying the interaction between tumor cells, extracellular matrix, and proteoglycans. Breast cancer exhibits a marked tropism for bone tissue, and in patients with metastatic disease, bone is one of the most frequent sites of dissemination. Recent population-based studies using computed tomography (CT) scans have estimated a high cumulative incidence of bone metastasis in breast cancer, second only to prostate cancer in several clinical analyses [2]. Sui et al. (2024) highlight that bone metastasis occurs in a substantial proportion of patients with advanced breast cancer and is associated with pain, pathological fractures, spinal cord compression, hypercalcemia, and impaired quality of life [9].

In breast cancer, disseminated tumor cells can colonize bone, remain dormant for years, and later reactivate to cause predominantly osteolytic metastases, although mixed lesions also occur [122,124]. These cells secrete PTHrP, IL-11, IL-6, TNF-α, CXCLs, TGF-β, RANKL, Jagged1, and MMPs, which promote osteoclast differentiation and activation [122,128,129,130,174]. PTHrP enhances osteoblast RANKL expression through cyclic adenosine monophosphate/protein kinase A/cAMP response element-binding protein (cAMP/PKA/CREB) signaling, and RANKL then binds RANK on osteoclast precursors to drive bone resorption [130]. This resorption releases matrix-stored TGF-β, IGFs, BMPs, PDGF, and FGF, further supporting tumor growth and amplifying the tumor–bone vicious cycle [128,130].

Proteoglycans contribute to breast cancer bone metastasis as structural components of the bone extracellular matrix and tumor glycocalyx and as signaling regulators of tumor–bone crosstalk [17,95], as shown in Table 5. Heparan sulfate proteoglycans, including syndecans, glypicans, perlecan/HSPG2, agrin, and collagen XVIII, bind growth factors, cytokines, chemokines, morphogens, proteases, and extracellular matrix proteins through their sulfated glycosaminoglycan chains, thereby controlling ligand availability, receptor presentation, and gradient formation within the metastatic niche [94,159]. In this context, they modulate FGF/FGFR, VEGF/VEGFR, HGF/MET, WNT/β-catenin, TGF-β/SMAD, BMP, CXCL/CXCR, RANK–RANKL–OPG, IL-6/JAK–STAT3, and Notch/Jagged1 signaling, which collectively regulate tumor-cell adhesion, migration, invasion, angiogenesis, immune-cell recruitment, osteoclastogenesis, and survival in the bone microenvironment [52,94,119,136,159]. Bone matrix components also engage integrins, CD44, RHAMM, and syndecan-dependent co-receptor signaling, activating downstream FAK/Src, PI3K/AKT, MAPK/ERK, Rho guanosine triphosphatase (Rho GTPase), and YAP/TAZ mechanotransduction programs that promote metastatic colonization [128,136,205]. During tumor progression, extracellular matrix remodeling mediated by MMPs, heparanase, sulfatases, hyaluronidases, and other proteases cleaves proteoglycan core proteins or modifies glycosaminoglycan sulfation patterns, releasing matrix-bound ligands and generating bioactive fragments that further enhance growth-factor signaling, chemotaxis, invasion, and the tumor–bone vicious cycle [35,94].

Among the most relevant proteoglycans in breast cancer are versican, syndecans, perlecan/HSPG2, biglycan, decorin, lumican, serglycin, glypicans, and the HA-CD44/RHAMM axis [19,42]. Versican is particularly important because its G3 domain has been associated with increased motility, invasion, EGFR/ERK, EGFR/JNK, and AKT signaling, as well as alterations in osteoblastic growth and differentiation, which may promote the adaptation of breast cancer cells to the bone niche and tumor migration, EGFR-mediated signaling, and alterations in osteoblastic cells during bone metastasis of breast cancer [23,78].

The HA-CD44/RHAMM axis is another key regulator [31,209]. Although HA is not a strict proteoglycan, it acts as an essential component of the pericellular matrix and is functionally associated with hyalectans, such as versican. In breast cancer, this axis can promote extracellular matrix (ECM) remodeling, cellular plasticity, migration, therapeutic resistance, interactions with stromal cells, and immunomodulation [210,211]. Some studies indicate that these molecules can act as “friends or foes” of the tumor, depending on the tumor subtype, location, cell type, stage of progression, and state of matrix remodeling [19,209].

Decorin is a proteoglycan with predominantly antitumor functions [59,171]. Its ability to antagonize tyrosine kinase receptors, including EGFR, MET, ERBB2/HER2, and VEGFR2, as well as negatively modulate TGF-β, WNT/β-catenin, HIF-1α, and VEGFA, positions it as a molecule that suppresses proliferation, angiogenesis, and invasion [171]. In preclinical models, Yang et al. developed a decorin-expressing oncolytic adenovirus, Ad.dcn, and demonstrated that its systemic administration in nude mice with skeletal metastases derived from MDA-MB-231-luc cells significantly inhibited the progression of bone lesions [170]. This effect was accompanied by a lower tumor burden, reduced osteolytic destruction, fewer osteoclasts, and decreased hypercalcemia associated with bone destruction. Mechanistically, decorin expression reduced Met, β-catenin, and VEGFA, inhibited cell migration, and induced mitochondrial autophagy, suggesting a combined effect on tumor cells and the bone microenvironment [44,144,170]. However, the evidence remains preclinical, and its clinical translation still requires further validation.

## 7. Prostate Cancer Bone Metastasis

Prostate cancer is the paradigmatic model of osteoblastic bone metastasis, although it is now recognized that these lesions are not purely bone-forming. In advanced disease, bone involvement is very common and is associated with pain, fractures, spinal cord compression, treatment resistance, and mortality [126]. Recent clinical studies have indicated that prostate cancer bone metastasis shows a radiologically predominant osteosclerosis, but with the coexistence of osteoclastic activity, accelerated remodeling, and the formation of immature or structurally abnormal bone [125,126].

Bone metastasis in prostate cancer is a dynamic process involving colonization, dormancy, reactivation, and bone remodeling [126]. Tumor cells reach bone, attracted by chemotactic and adhesive signals from the bone microenvironment, including CXCL12/CXCR4, CCL2, RANKL, VEGF, and integrins. Once established, they can remain dormant near osteoblasts and stromal cells, resisting conventional therapies. Subsequently, factors such as TGF-β, IL-6, AKT, and ERK promote their reactivation, proliferation, and invasion. Ultimately, tumor cells disrupt the balance between osteoblasts and osteoclasts, promoting pathological bone remodeling that favors metastatic growth and contributes to the skeletal complications of prostate cancer [126]. Eltit et al. (2024) analyzed osteosclerotic bone metastasis from prostate cancer and showed that these lesions contain pathological bone with altered structural organization, supporting the idea that radiological sclerosis does not equate to functionally normal bone [125].

Proteoglycans play a central role in this form of metastasis by linking tumor signaling to desmoplastic remodeling, growth factor availability, and interactions with osteoblasts, osteoclasts, macrophages, and bone marrow fibroblasts [27]. Studies indicate that perlecan acts as a dynamic regulator of bone invasion. While intact perlecan and its IV-3 domain promote tumor cohesion and inactivate FAK, its cleavage by MMP-7 promotes FAK activation, cell dispersal, and a metastatic phenotype capable of crossing perlecan-rich barriers into the bone marrow [26]. Also, perlecan/HSPG2 promotes stromal remodeling and the availability of growth factors in prostate cancer metastases, while decorin may exert anti-metastatic effects by inhibiting tyrosine kinase receptors, angiogenesis, and osteoclastic activity [26,28,212].

Perlecan/HSPG2 is one of the most relevant proteoglycans in metastatic prostate cancer. Its location in basement membranes and the pericellular matrix allows it to act as a reservoir for FGF, VEGF, HGF, WNTs, and other bioactive factors [131,132,133]. In the desmoplastic prostatic microenvironment, NF-κB activation can increase *HSPG2* expression; furthermore, MMP-7/matrilysin-mediated cleavage of perlecan can alter its complexes with semaphorin 3A and promote FAK-mediated stromal invasion [26,28,161]. This perlecan–MMP-7–Sema3A–FAK axis is a particularly relevant mechanism for the transition to tissue invasion and advanced bone disease [26,161].

Another axis corresponds to SULF1/SULF2–heparan sulfate–WNT. Sulfases modify the 6-O-sulfation patterns of heparan sulfate and, consequently, the availability of ligands such as WNT, FGF, and VEGF [175,176]. In three-dimensional models of prostate cancer bone metastasis integrating tumor cells, stroma, and macrophages, SULF1 has been associated with the regulation of WNT3A-mediated growth in perlecan-modified matrices [27]. SULF1 emerges as a negative regulator of Wnt3A-induced growth in perlecan-modified matrices. In this biomimetic system, perlecan/HSPG2 acts as a reservoir of heparan sulfate-bound growth factors, including Wnt3A, thus promoting the local availability of proliferative signals for tumor cells. However, the SULF1 enzyme, produced primarily by bone stromal fibroblasts, removes the 6-O-sulfate groups from the heparan sulfate chains, limiting the ability of these proteoglycans to stabilize Wnt3A and facilitate the formation of signaling complexes on the cell surface. Importantly, the loss of *SULF1* in stromal fibroblasts increased tumor cellularity and the size of C4-2B cell tumoroids in the presence of Wnt3A, indicating that *SULF1* acts as a molecular “brake” on Wnt3A signaling in the bone metastatic niche. These findings position the perlecan-heparan sulfate-SULF1-Wnt3A axis as a contextual matrix remodeling mechanism that can modulate tumor progression in prostatic bone metastasis, even in the presence of tumor-associated macrophages with an M2-like phenotype [27,103].

The beta-glycan/TGFβ-RIII–p38MAPK–RB axis is particularly relevant for tumor dormancy. In this context, differentiated osteoblasts secrete factors such as TGFβ2 and GDF10, which activate TGFβ-RIII/betaglycan in tumor cells [83,179]. This signaling induces activation and nuclear translocation of p38 mitogen-activated protein kinase (p38MAPK), which phosphorylates RB at the S249/T252 sites, thereby reinforcing cell-cycle arrest and promoting a quiescent or latent state [83,180]. Thus, this axis links signals from the bone niche to the inhibition of tumor proliferation, explaining why some disseminated cells can remain viable yet dormant for years before reactivating and generating clinically evident bone metastases. [83]. This mechanism is important because it links cell-surface proteoglycans, TGF-β signaling, and cell-cycle control in disseminated tumor cells.

Furthermore, proteoglycans contribute to therapeutic resistance in prostate cancer by modulating the extracellular matrix, tumor signaling, cellular plasticity, EMT, stemness, and microenvironmental interactions (Table 6). Proteoglycans such as ASPN/asporin, syndecan-1, and versican are associated with docetaxel resistance; betaglycan/TGFβ-RIII and biglycan are associated with a poor response to hormonal therapies and progression to castration-resistant prostate cancer; SPOCK1/testican-1 is involved in enzalutamide resistance; and perlecan/HSPG2 promotes radioresistance [16,180]. Therefore, these proteoglycans can be considered candidate biomarkers or investigational targets, with variable and often indirect bone-specific validation in advanced prostate cancer.

## 8. Other Cancers and Bone-Tropic Disease

Although breast and prostate cancers account for the majority of the literature on bone metastases, other tumors also exhibit a significant tropism for bone [124]. These include lung, kidney, and thyroid cancers, hepatocellular carcinoma, melanoma, multiple myeloma, and some primary bone tumors or sarcomas with pulmonary or bone spread [213,214], Table 7. A recent population-based study, based on natural language processing of CT reports, found cumulative incidences of bone metastasis not only in prostate and breast cancers, but also in lung, melanoma, hepatobiliary, endocrine/thyroid, genitourinary, and pancreatic tumors [2].

In these tumors, the specific evidence on proteoglycans in bone metastasis is more heterogeneous and less mature than in breast or prostate cancer. However, several patterns are relevant. First, many osteotropic tumors use common homing and colonization axes, including CXCL12/CXCR4, integrins, osteopontin, VCAM-1, TGF-β, RANKL, and MMPs [50,106,215]. Second, HSPGs and heparanase are cross-cutting regulators of invasion, angiogenesis, and growth factor release [38,216]. Third, some tumors show particular associations: lumican in bone metastasis from lung cancer, syndecan-1/CD138 in multiple myeloma, glypicans in hepatocellular carcinoma and melanoma, and syndecan-4/fibronectin or lumican/decorin in primary bone tumors [50,217,218,219].

In lung cancer, bone metastasis is clinically relevant due to its association with pain, skeletal events, and poor prognosis. LUM, for example, has been identified as an SLRP with a prometastatic function in bone. Its expression is increased in lung cancer cells selected for their bone tropism, and its silencing reduces adhesion to extracellular matrix components, migration, invasion, and the formation of bone metastasis in vivo [172]. Also, lumican secreted by the tumor cells themselves can act autocrinally, promoting FAK activation, MMP2/9-mediated proteolytic activity, and early seeding in the bone marrow. This finding demonstrates that SRPs are not necessarily antitumor in all contexts, but rather can acquire prometastatic functions depending on the tumor type, matrix composition, and target niche [41,142,173].

In multiple myeloma, the disease is not considered a solid tumor metastasis but rather a bone disease highly dependent on the bone marrow niche. In hematologic malignancies, the concept of a “malignant hematopoietic proteoglycome” describes the network of proteoglycans expressed by normal hematopoietic cells, malignant cells, and components of the bone marrow microenvironment. These proteoglycans act as multivalent regulators by integrating signals for adhesion, cell trafficking, differentiation, survival, matrix remodeling, and therapeutic response. In normal bone marrow, molecules such as perlecan, agrin, decorin, biglycan, versican, CD44, syndecans, glypicans, and NG2/CSPG4 contribute to the architecture and function of the hematopoietic niche. In malignant conditions, its aberrant or ectopic expression can promote bone marrow infiltration, tumor-stroma interactions, angiogenesis, osteolysis, immune evasion, and drug resistance, as occurs with CD44 in leukemias, syndecan-1/CD138 in multiple myeloma, and NG2/CSPG4 in leukemia subtypes associated with genetic rearrangements [193,194].

In hepatocellular carcinoma and melanoma, direct evidence of bone metastasis is more limited, but data link glypican-3/GPC3 to circulating tumor cells in hepatocellular carcinoma and glypican-6/GPC6 to metastatic progression in melanoma [220,221,222]. In primary bone tumors, such as osteosarcoma or giant cell tumor, alterations in syndecan-4, fibronectin, lumican, and decorin have been described [223,224,225,226], suggesting that proteoglycans also play a role in the biology of tumors originating in the bone compartment.

**Table 7:** Proteoglycan/GAG/ECM axes in other bone-tropic solid tumors and comparative bone-niche diseases.

Tumor or Disease	Main PG/GAG/ECM Axis	Dominant Functional Contribution	Evidence Directness	Translational Interpretation	Ref.
Lung cancer	Lumican, HSPGs, HPSE, HA-CD44, MMPs	Lumican promotes adhesion, migration, invasion, FAK activation, MMP2/9 activity, and bone metastatic seeding. HSPG/HPSE axes may support angiogenesis and growth-factor release.	Direct for lumican; mixed/extrapolated for other axes.	Lumican is the strongest non-breast/non-prostate bone-metastasis example; other axes require validation.	[2,172,227]
Renal cell carcinoma	HSPGs, perlecan/HSPG2, HPSE, VEGF-associated ECM	May support angiogenesis, vascular invasion, matrix remodeling, and tumor–stroma interaction.	Mostly extrapolated.	PG-specific mechanisms remain insufficiently validated in renal cancer bone metastasis.	[3,17]
Thyroid and other endocrine cancers	HSPGs, HA-CD44, integrins, MMPs	May regulate adhesion, ECM remodeling, vascularization, and local invasion.	Extrapolated.	Should be presented as a plausible ECM-related mechanism, not as a proven PG-driven bone-metastasis axis.	[2,14]
Hepatocellular carcinoma	GPC3, HSPGs, HPSE	GPC3 may contribute to dissemination through WNT/β-catenin, FGF-related signaling, and HSPG-dependent tumor–stroma interaction.	Extrapolated for bone metastasis.	GPC3 is relevant in HCC biology but not validated as a bone-metastasis-specific biomarker.	[2,220]
Melanoma	GPC6, HSPGs, HA-CD44, MMPs	May contribute to metastatic progression, invasion, migration, and ECM remodeling.	Extrapolated for bone metastasis.	GPC6 should be discussed as a metastatic-progression marker, not as a validated skeletal-tropism mechanism.	[2,220,221,222]
Multiple myeloma	SDC1/CD138, HPSE, HSPGs, perlecan, decorin, biglycan.	Regulates myeloma–bone marrow adhesion, stromal interaction, growth-factor signaling, angiogenesis, osteolysis, and drug resistance.	Direct for bone marrow disease; comparative for solid-tumor bone metastasis.	Useful as a comparative PG-rich bone-niche disease, but not equivalent to solid-tumor metastasis.	[2,162,220,228]
Osteosarcoma and primary bone tumors	SDC4, fibronectin, lumican, decorin, HSPGs, MMPs	Regulate adhesion, ECM organization, migration, differentiation, invasion, and matrix-dependent signaling.	Direct for primary bone tumor biology; not direct for bone metastasis.	Useful for comparison of bone-matrix biology; should be clearly separated from metastatic solid tumors.	[142,223,226]
Giant cell tumor of bone	Decorin, lumican, collagen-associated ECM, MMPs	Altered ECM composition may contribute to osteolysis, local aggressiveness, and rare metastatic potential.	Direct for primary bone tumor biology; comparative only.	Not evidence of solid-tumor bone metastasis. Decorin/lumican should not be considered validated skeletal-metastasis biomarkers or therapeutic targets.	[223]

Abbreviations: ECM, extracellular matrix; GAG, glycosaminoglycan; GPC3, glypican-3; GPC6, glypican-6; HA, hyaluronan; HCC, hepatocellular carcinoma; HPSE, heparanase; HSPG, heparan sulfate proteoglycan; MMP, matrix metalloproteinase; PG, proteoglycan; SDC1/CD138, syndecan-1/cluster of differentiation 138; SDC4, syndecan-4; VEGF, vascular endothelial growth factor. Evidence directness was classified as follows: direct evidence, when derived from patient bone metastasis samples or In Vivo skeletal metastasis models; mixed evidence, when both bone-specific and extrapolated studies support the same axis; extrapolated evidence, when derived mainly from primary tumors, non-bone metastatic sites, or general cancer models; and comparative evidence, when derived from multiple myeloma or primary bone tumors and used only to contextualize PG/GAG/ECM remodeling in the bone niche.

## 9. Clinical and Translational Relevance

### 9.1. Current Clinical Management and Unmet Needs

Bone metastasis remains a major clinical challenge because current therapeutic strategies rarely eradicate established skeletal disease and are often aimed at preventing or delaying skeletal-related events, reducing pain, preserving neurological function, and maintaining quality of life. Current management includes systemic therapies directed against the primary tumor, radiotherapy, orthopedic or neurosurgical intervention in selected cases, radiopharmaceuticals, bisphosphonates, denosumab and supportive care [3,4]. Treatment selection depends on tumor type, extent of skeletal involvement, pain, neurological risk, mechanical instability, prognosis, and the overall therapeutic objective [3,4,106,215]. These approaches do not fully address the biological complexity of the metastatic bone niche, which is shaped by tumor cells, osteoblasts, osteoclasts, osteocytes, immune cells, adipocytes, endothelium, and the extracellular matrix [3,48,106,138]. This network regulates homing, dormancy, reactivation, bone remodeling, angiogenesis, and immunosuppression; therefore, future strategies should combine tumor control, preservation of bone architecture, and modulation of the metastatic niche. Therefore, proteoglycans are clinically relevant because they connect tumor-intrinsic signaling with bone remodeling, extracellular matrix organization, immune regulation, angiogenesis, dormancy, and therapy resistance [37,75,142,143]. From this perspective, proteoglycans should not be viewed only as structural matrix components, but as dynamic regulators of the tumor–bone–immune–matrix interface. Their translational value can be organized into three major areas: biomarkers and niche-stratification tools, therapeutic targets, and modulators of treatment response.

### 9.2. Proteoglycans as Biomarkers and Niche-Stratification Tools

Proteoglycans may provide information that is complementary to conventional biomarkers. Whereas classical biomarkers often reflect tumor lineage, receptor status, genomic alterations, or tumor proliferation, proteoglycans can inform on the functional state of the extracellular matrix, including matrix stiffness, ligand sequestration, adhesion, invasion, angiogenesis, inflammatory remodeling, immunomodulation, and therapeutic resistance [37,75,142]. Their core proteins, glycosaminoglycan chains, sulfation patterns, soluble ectodomains, and proteolytic fragments may reflect ongoing matrix remodeling and tumor–stroma communication [42,142,144]. Several analytical sources could be considered for proteoglycan-based biomarker development, including primary tumor samples, bone metastasis biopsies, circulating tumor-associated fragments, extracellular vesicles, serum or plasma proteoglycans, and proteolysis-derived matrikines [142,143]. For example, SDC1/CD138 has established value in multiple myeloma [187,188,189]. Circulating SDC1 has also been associated with chemotherapy resistance in castration-resistant prostate cancer [186]. HSPG2/perlecan and its fragments have been proposed as indices of invasion in prostate cancer [26]. In breast cancer, proteoglycan-expression signatures may help distinguish aggressive phenotypes and matrix-remodeled tumors [25]. This context-dependent plasticity suggests that proteoglycans may function not merely as tumor-associated biomarkers but also as dynamic indicators of tumor microenvironment status, metastatic niche remodeling, matrix organization, and treatment vulnerability.

### 9.3. Therapeutic Targeting of Proteoglycan-Regulated Axes

From a therapeutic standpoint, proteoglycan-regulated pathways offer several potential intervention points. These include enzymatic remodeling of heparan sulfate by heparanase/HPSE, extracellular editing of heparan sulfate sulfation by SULF1/SULF2, HA-CD44/RHAMM-dependent signaling, syndecan-mediated co-receptor activity, perlecan/HSPG2-dependent growth-factor storage, MMP- and cathepsin-mediated proteolysis, LOX/LOXL2-mediated matrix stiffening, integrin–FAK/Src mechanotransduction, and decorin-dependent antagonism of receptor tyrosine kinase signaling [22,31,103,142,144,175,183,190]. Heparanase inhibition may reduce the release of heparan sulfate-bound growth factors, chemokines, and morphogens, thereby limiting angiogenesis, invasion, osteolytic signaling, and metastatic niche remodeling [34,102,144,229,230]. Modulation of SULF1/SULF2 activity could alter heparan sulfate-dependent signaling gradients and modify the availability of WNT, FGF, VEGF, and other ligands within the metastatic microenvironment [35,103,113,143,175,176]. Targeting the HA-CD44/RHAMM axis may reduce tumor-cell plasticity, migration, stemness-associated programs, and resistance to therapy, especially in HA-rich and inflammatory tumor niches [31,97,98,209]. Decorin-based approaches are also attractive because decorin can antagonize multiple receptor tyrosine kinases and suppress pro-angiogenic, pro-invasive, and pro-metastatic signaling pathways [59,79,170,171,231].

In addition, extracellular matrix-directed strategies may improve the effectiveness of conventional therapies by modifying mechanical barriers, proteolytic remodeling, and drug penetration. Current evidence indicates that the extracellular matrix is not only a physical scaffold, but also an active regulator of metastatic dissemination, therapeutic resistance, and immune exclusion [15,143,182,183,184]. Therefore, proteoglycan-targeted strategies are likely to be most effective when combined with tumor-directed therapy, antiresorptive agents, radiotherapy, radiopharmaceuticals, immunotherapy, or matrix-normalizing interventions rather than used as isolated treatments.

### 9.4. Integration with Immunotherapy

The integration of proteoglycan biology with immunotherapy is particularly relevant in bone metastasis. The bone marrow is an immune-rich microenvironment containing macrophages, T lymphocytes, NK cells, dendritic cells, MDSCs, and neutrophils, and other immune populations that can either restrict or promote metastatic progression depending on their activation state and spatial organization [13,61,63,69,232]. Proteoglycans and GAG-rich matrices can influence this immune landscape by regulating chemokine availability, cytokine gradients, macrophage polarization, myeloid-cell recruitment, lymphocyte infiltration, and matrix stiffness [70,74,75,76,110]. This has therapeutic implications. Immune checkpoint inhibitors, anti-TGF-β approaches, MDSC-directed therapies, macrophage-targeted strategies, bispecific antibodies, and chimeric antigen receptor (CAR) T cell therapies may be affected by the physical and biochemical properties of the metastatic matrix [68,84,233,234]. Matrix stiffness, HA-rich barriers, proteoglycan fragments, and heparan sulfate-dependent chemokine presentation may contribute to immune exclusion or immunosuppressive niche formation [70,71,72,73,75,143].

### 9.5. Challenges for Clinical Translation

Despite their translational potential, several challenges limit the immediate clinical application of proteoglycan-based strategies. First, proteoglycans are pleiotropic and highly context-dependent molecules. The same proteoglycan may promote tumor progression in one tumor subtype or metastatic niche, suppress tumor growth in another, or change function according to glycosylation, sulfation, proteolytic processing, cellular source, and matrix organization [15,37,42,142,182]. Second, many proteoglycans and matrix-remodeling enzymes have essential physiological functions in bone, vasculature, hematopoiesis, and immune regulation; therefore, non-selective systemic inhibition may cause toxicity or interfere with tissue homeostasis [144,182]. Third, most current evidence derives from in vitro systems, xenografts, engineered three-dimensional cultures, or indirect biomarker studies, whereas validation in patient-derived bone metastasis specimens remains limited [48,138,195,196,197]. For these reasons, the most realistic translational strategy is not broad inhibition of entire proteoglycan families, but context-specific targeting based on tumor type, lesion phenotype, matrix state, immune composition, and proteoglycan/GAG signatures. Future studies should integrate matrisome profiling, spatial proteomics, single-cell and spatial transcriptomics, GAGomics, degradomics, extracellular-vesicle analysis, and mineralized three-dimensional bone niche models to identify clinically actionable proteoglycan-dependent vulnerabilities [182,198,199,200,201,202,203,204]. Such approaches may help stratify patients according to metastatic niche biology and guide the rational combinations of tumor-directed therapy, bone-targeted treatment, immunotherapy, and matrix-modulating interventions.

## 10. Knowledge Gaps and Future Directions

Despite increasing evidence that proteoglycans regulate bone metastatic progression, several conceptual, technical, and translational gaps remain. A major knowledge gap is the lack of integrated spatial maps of the metastatic bone matrix. Most studies focus on one or a few proteoglycans and rarely integrate core protein identity, GAG class, sulfation pattern, proteolytic fragmentation, cellular source, and spatial location within osteolytic, osteoblastic, or mixed lesions. This is important because extracellular matrix function depends not only on molecular composition, but also on architecture, mechanics, proteolysis, and tissue organization [182]. Therefore, future studies should define proteoglycan networks as spatially organized matrix systems rather than as isolated molecules.

Furthermore, there is limited characterization of proteoglycans in human bone metastasis samples. Bone biopsies are difficult to obtain and process, decalcification can affect molecular analyses, and many studies rely on in vitro models or xenografts. Integrated analyses of single-cell RNA-seq (scRNA-seq), spatial transcriptomics, spatial proteomics, glycomics, GAGomics, degradomics, and insoluble matrix proteomics are needed to determine which proteoglycans are produced by tumor cells, osteoblasts, osteoclasts, osteocytes, fibroblasts, endothelial cells, or immune populations [19,198,199,200,201,202,203,204].

Another unresolved issue is the distinction between association and causality. Many studies link proteoglycans to metastasis through differential expression or correlative biomarker analyses, but fewer studies demonstrate whether these molecules actively drive bone colonization, dormancy, reactivation, immune escape, or therapy resistance [37,42,70]. To address this, functional models should incorporate tumor cells together with osteoblasts, osteoclasts, osteocytes, endothelial cells, macrophages, neutrophils, lymphocytes, and native or biomimetic extracellular matrix. Mineralized three-dimensional cultures, bone organoids, bone-on-a-chip systems, and multicellular co-cultures may be particularly useful for testing axes such as perlecan/HSPG2, SULF1/SULF2, HA-CD44/RHAMM, and desmoplastic remodeling under controlled but physiologically relevant conditions [195,197,235,236].

The proteoglycan–immune interface in bone metastasis also remains incompletely understood. Proteoglycans such as versican and biglycan can function as inflammatory matrix mediators, HSPGs and heparanase can regulate chemokine and growth-factor availability, and matrix stiffness can promote immune exclusion or favor a suppressive myeloid phenotype [71,72,73,74,76]. Although ECM immunoregulation is increasingly recognized as a determinant of immune evasion and immunotherapy response, the specific contribution of proteoglycan/GAG remodeling to immune-cell positioning, activation, exclusion, or suppression in metastatic bone lesions remains poorly defined [73,75,94,143,159]. Future studies should integrate spatial immune profiling with proteoglycan/GAG mapping to determine whether matrix-targeted interventions can improve antitumor immunity in bone metastasis.

Another critical gap concerns the role of proteoglycan remodeling in dormancy and metastatic reactivation. Osteoblastic factors and members of the TGF-β family, such as TGFβ2 and GDF10, can induce dormancy of prostate cancer cells through TGFβRIII–p38MAPK–RB [83,180,181,237]. However, it remains unclear how matrix stiffness, GAG sulfation, proteoglycan fragmentation, heparanase activity, HA-CD44/RHAMM signaling, inflammatory remodeling, or osteoclast-dependent matrix turnover modify the switch between quiescence and metastatic outgrowth. This question is clinically relevant because skeletal relapse may occur years after treatment of the primary tumor, suggesting that the matrix may act not only as a scaffold for disseminated tumor cells but also as a regulator of long-term dormancy, survival, and reactivation.

Finally, future research should move toward niche-informed precision medicine. This will require biomarkers that integrate imaging, serum bone turnover markers, ECM signatures, circulating proteoglycans or proteoglycan fragments, extracellular vesicle cargo, immune profiling, and tumor genomic data. Such integrated strategies could help classify bone metastases according to lesion phenotype, matrix state, immune composition, dormancy potential, and treatment vulnerability. From a therapeutic perspective, rational combinations should be explored, including antiresorptive agents or radiopharmaceuticals with ECM-modulating therapies, immunotherapy with TGF-β, heparanase, or HA-CD44/RHAMM modulators, and systemic therapies with matrix-normalization strategies [22,31,106,143,183,190,229,230]. Therefore, the strategy should not involve eliminating the matrix but rather reprogramming the metastatic niche to be less prone to colonization, immunosuppression, reactivation, and therapy resistance. To organize these priorities, Table 8 summarizes the major knowledge gaps, current limitations, proposed research approaches, and expected translational impact of future proteoglycan-focused studies in bone metastasis.

## 11. Conclusions

Proteoglycans are emerging as functional components of the bone metastatic niche, capable of integrating signals from the extracellular matrix, tumor cells, the bone compartment, and the immune system. Beyond their structural functions, these molecules modulate key processes involved in metastatic progression, including extracellular matrix remodeling, tumor–bone crosstalk, osteolytic and osteoblastic niche formation, angiogenesis, immune evasion, metastatic dormancy, reactivation, and therapy resistance.

The evidence reviewed here indicates that proteoglycan function is highly context dependent. Their biological impact varies according to tumor type, stage of progression, cellular source, glycosaminoglycan composition, sulfation patterns, ectodomain shedding, and enzymatic remodeling of the metastatic microenvironment. This complexity prevents their interpretation as a homogeneous molecular family and supports their analysis as dynamic signaling networks rather than isolated structural components.

Direct evidence of bone metastasis is strongest for selected proteoglycan/GAG-associated axes, including versican and decorin in breast cancer models; perlecan/HSPG2 and MMP-7–Sema3A–FAK signaling in prostate cancer; SULF1/SULF2-dependent heparan sulfate remodeling in bone-niche models; and TGFβ-RIII/betaglycan-associated dormancy signaling. In contrast, several proposed roles of proteoglycan expression, GAG sulfation, heparanase activity, HA-CD44/RHAMM signaling, proteoglycan shedding, and matrix-derived fragments in dormancy maintenance, reactivation, immune escape, and therapy resistance are incompletely validated across tumor types.

Therefore, proteoglycans offer opportunities for biomarker discovery, patient stratification, and therapeutic targeting of the bone metastatic niche. However, their clinical application requires validation in human samples, more representative experimental models of the bone microenvironment, and studies capable of distinguishing descriptive associations from causal mechanisms. A deeper understanding of how proteoglycans regulate interactions among tumor cells, bone, immunity, and extracellular matrix may facilitate new strategies to prevent bone colonization, limit metastatic progression, overcome dormancy-associated relapse, and optimize combination therapies targeting both tumor cells and the supportive metastatic niche. Future studies should integrate matrisome profiling, spatial proteomics, single-cell and spatial transcriptomics, glycosaminoglycan omics, degradomics, extracellular vesicle analysis, and three-dimensional bone niche models. These approaches are essential to identify which proteoglycan alterations are causal drivers of skeletal metastasis, which are biomarkers of niche state, and which can be safely targeted for therapeutic benefit.

## Figures and Tables

**Table 1:** Proteoglycans and associated GAG/ECM axes relevant to tumor progression and bone metastasis.

Proteoglycan or GAG/ECM-Associated Axis	Main GAG Chains	Key Structural Feature	Main Signaling Pathways	Metastatic Functions	Bone Metastasis Relevance	Ref.
Syndecan-1	Heparan sulfate/chondroitin sulfate	Ectodomain shedding	EGFR, FAK, Src, WNT	Migration, invasion, immune modulation, therapy resistance	Osteoclast activation, tumor–bone interaction	[16,19]
Syndecan-4	Heparan sulfate	Focal adhesion/cytoskeletal regulation	FAK, PKCα, integrins	Adhesion, migration, and mechanotransduction	Bone niche colonization	[16]
Perlecan/HSPG2	Heparan sulfate	Growth factor reservoir in basement membrane/ECM	FAK, WNT, VEGF, FGF	Stromal invasion, angiogenesis, growth factor availability	Desmoplastic remodeling of bone metastatic niche	[26,27]
Versican	Chondroitin sulfate	Large ECM proteoglycan; G3 domain	EGFR, ERK, AKT, TGF-β	Migration, invasion, tumor plasticity	Osteoblastic dysfunction, bone microenvironment remodeling	[19,23]
Decorin	Dermatan sulfate/chondroitin sulfate	Small leucine-rich proteoglycan; RTK antagonist	EGFR, MET, VEGFR2, TGF-β	Anti-migratory, anti-angiogenic, anti-metastatic	Inhibition of osteoclast-associated remodeling	[19,24]
Biglycan	Dermatan sulfate/chondroitin sulfate	Damage-associated molecular pattern-like activity	TLR2/4, NF-κB, TGF-β	Inflammation, invasion, angiogenesis	Bone inflammation and remodeling	[14,19]
Glypicans	Heparan sulfate	GPI-anchored membrane proteoglycans	WNT, Hedgehog, FGF	Proliferation, migration, stemness	Tumor–stroma signaling	[16,17]
SPOCK family	Heparan/chondroitin sulfate	Secreted matricellular proteoglycans	PI3K/AKT, WNT/β-catenin, EMT pathways	EMT, invasion, therapeutic resistance	Metastatic progression	[16]
Associated free GAG axis, not a proteoglycan: HA-CD44/RHAMM (CD168)	HA-associated complexes	Non-sulfated GAG-dependent receptor signaling	CD44, RHAMM/CD168, ERK, PI3K/AKT	Migration, stemness, immune evasion, therapy resistance	Colonization, latency and progression	[16,29]

Abbreviations: AKT, protein kinase B; CD44, cluster of differentiation 44; RHAMM(CD168), receptor for hyaluronan-mediated motility; ECM, extracellular matrix; EGFR, epidermal growth factor receptor; EMT, epithelial–mesenchymal transition; ERK, extracellular signal-regulated kinase; FAK, focal adhesion kinase; FGF, fibroblast growth factor; GAG, glycosaminoglycan; GPI, glycosylphosphatidylinositol; HA, hyaluronan; HSPG2, heparan sulfate proteoglycan 2/perlecan; MET, mesenchymal–epithelial transition factor receptor; NF-κB, nuclear factor kappa B; PI3K, phosphoinositide 3-kinase; PKCα, protein kinase C alpha; RTK, receptor tyrosine kinase; Src, proto-oncogene tyrosine-protein kinase Src; TGF-β, transforming growth factor beta; TLR, Toll-like receptor; VEGF, vascular endothelial growth factor; VEGFR2, vascular endothelial growth factor receptor 2; WNT, wingless/integrated signaling pathway.

**Table 2:** Structural alterations of proteoglycans associated with metastatic competence.

Structural Alteration	Mechanism	Biological Consequence	Examples	Ref.
Increased GAG sulfation	Enhances electrostatic binding to growth factors, chemokines and morphogens	Increased signaling intensity, migration, angiogenesis, and metastatic fitness	Heparan sulfate proteoglycans	[17,21]
Altered glycosylation	Modifies ligand binding, receptor clustering, and ECM interactions	Tumor plasticity, adhesion changes, and immune modulation	Syndecans, glypicans, perlecan	[14,17]
Ectodomain shedding	Proteolytic cleavage releases soluble proteoglycan fragments	Soluble signaling platforms that promote invasion, angiogenesis, and immune escape	Syndecan-1	[16,17]
Heparanase-mediated remodeling	Cleaves heparan sulfate chains and releases matrix-bound factors	ECM degradation, growth factor release, angiogenesis, and metastatic dissemination	Perlecan, syndecans, HSPGs	[17,22]
MMP-mediated cleavage	Proteolytic processing of ECM proteoglycans	Matrix remodeling, stromal invasion, and exposure of bioactive fragments	Perlecan, versican	[20,26]
Accumulation of ECM proteoglycans	Creates a dense, desmoplastic, and mechanically altered matrix	Tumor stiffness, invasion, altered mechanotransduction, and therapy resistance	Perlecan, versican, biglycan	[14,27]
Loss of tumor-suppressive proteoglycans	Reduced inhibition of receptor tyrosine kinase signaling	Increased proliferation, migration, angiogenesis, and metastatic potential	Decorin	[19,24]
*HA enrichment	Increases CD44/RHAMM (CD168)-dependent signaling	Stemness, migration, immune evasion, latency, and therapeutic resistance	Hyaluronan/CD44/RHAMM (CD168) axis	[16,29]

Abbreviations: CD44, cluster of differentiation 44; RHAMM(CD168), receptor for hyaluronan-mediated motility; ECM, extracellular matrix; GAG, glycosaminoglycan; HA, hyaluronan; HSPGs, heparan sulfate proteoglycans; MMP, matrix metalloproteinase. *HA is not a proteoglycan, but a free non-sulfated GAG functionally associated with hyalectans and CD44/RHAMM receptors [31].

**Table 4:** Convergent proteoglycan/GAG-regulated signaling axes in bone metastasis.

Regulatory Layer/Proteoglycan Axis	Main Proteoglycans or GAG-Associated Components	Remodeling Enzymes or Modifiers	Convergent Signaling Axes	Dominant Bone-Metastasis Outputs	Representative Tumor Contexts	Ref.
Cell-surface HSPG axis	Syndecans, glypicans, heparan sulfate chains	HPSE, SULF1/2, MMPs, ectodomain shedding	FGF/FGFR, VEGF/VEGFR, HGF/MET, EGF/EGFR, WNT/β-catenin, CXCL12/CXCR4, integrins–FAK/Src	Adhesion, invasion, angiogenesis, therapy resistance.	Breast cancer, prostate cancer, multiple myeloma, hepatocellular carcinoma, melanoma	[17,37,42,94,143,144,157,158,159]
Basement-membrane/pericellular HSPG axis	Perlecan/HSPG2, collagen XVIII/endostatin, agrin	HPSE, MMP-7, MMP14, cathepsins, SULF1/2	FAK/Src, VEGF/VEGFR, FGF/FGFR, HGF/MET, WNT/BMP, TGF-β/SMAD	Stromal invasion, desmoplasia, growth-factor storage.	Prostate cancer, breast cancer, matrix-rich and desmoplastic bone metastases	[26,27,28,40,95,107,131,132,133,137,161]
HA–hyalectan pericellular axis	HA, versican/VCAN, CD44, RHAMM/CD168, hyalectans	HYAL1/2, HAS2, MMPs, ADAMTS proteases	CD44/RHAMM, EGFR/ERK, EGFR/JNK, PI3K/AKT, ROCK1, *ZEB1/HAS2*, NF-κB, TGF-β-associated signaling	Motility, plasticity, inflammation, reactivation.	Breast cancer, prostate cancer, lung cancer, aggressive HA-rich tumors	[23,31,45,76,97,98,99,110,162,163,164,165,166,167,168]
SLRP/collagen-remodeling axis	Decorin, biglycan, lumican, collagen-associated SLRPs	MMPs, cathepsins, LOX/LOXL2, inflammatory proteases	EGFR, ERBB2/HER2, MET, VEGFR2, TGF-β/SMAD, WNT/β-catenin, TLR2/4–NF-κB, integrins–FAK/Src	Collagen remodeling, immune activation, metastasis modulation.	Breast cancer, prostate cancer, lung cancer, primary bone tumors	[37,41,42,44,59,74,75,79,100,169,170,171,172,173]
Proteoglycan-regulated osteolytic niche axis	HSPGs, syndecan-1/CD138, perlecan/HSPG2, versican, HA-CD44/RHAMM, bone matrix PGs	HPSE, MMPs, HYAL1/2, cathepsins, ADAMTS	RANKL/RANK/OPG, PTHrP/GLI2, TGF-β/SMAD, IGF/IGF1R, BMPs, CXCL12/CXCR4, IL-6/JAK–STAT3, TNF-α/NF-κB	Osteolysis, growth-factor release, vicious cycle.	Breast cancer, multiple myeloma, lung cancer, renal cancer, mixed lesions	[5,22,52,53,54,94,119,122,124,127,128,129,130,174]
Proteoglycan-regulated osteoblastic/desmoplastic niche axis	Perlecan/HSPG2, glypicans, syndecan-4, SULF-modified HSPGs, decorin, biglycan, HA-associated matrix	SULF1/2, MMP-7, LOX/LOXL2, MMPs, cathepsins	WNT/BMP, FGF/FGFR, VEGF/VEGFR, HGF/MET, integrins–FAK/Src, TGF-β/SMAD, NF-κB	Aberrant osteogenesis, desmoplasia, matrix stiffening.	Prostate cancer, osteoblastic/mixed bone metastases	[26,27,28,35,60,95,103,113,117,125,126,131,132,133,137,161,175,176]
Bone homing and metastatic niche-retention axis	HSPGs, HA-CD44/RHAMM, osteopontin-associated PG/GAG complexes, integrin-associated matrix PGs	HPSE, MMPs, SULF1/2, matrix proteases	CXCL12/CXCR4, VCAM-1/α4β1, osteopontin–integrins/CD44, integrins β1/β3–FAK/Src, RANKL/RANK	Bone homing, retention, colonization, early dormancy.	Breast cancer, prostate cancer, lung cancer, multiple bone-tropic tumors	[50,51,92,122,124,138,139,140,177,178]
Dormancy/reactivation axis	Betaglycan/TGFβRIII, perlecan/HSPG2, HA-CD44/RHAMM, HSPGs, osteoblast-derived matrix PGs	HPSE, SULF1/2, MMPs, HYAL1/2, osteoclast-associated proteases	TGFβ2/TGFβRIII–p38MAPK–RB, BMPs, CXCL12/CXCR4, integrins–FAK/Src, ERK/p38 balance, NF-κB	Quiescence, reactivation, late recurrence.	Prostate cancer, breast cancer, multiple myeloma	[80,81,82,83,84,85,86,101,121,179,180,181]
Immune–ECM remodeling axis	Versican, biglycan, HSPGs, HA-CD44/RHAMM, perlecan/HSPG2, matrix-associated PG fragments	HPSE, SULF1/2, MMPs, ADAMTS, cathepsins, cytokine-induced remodeling	TLR2/4–NF-κB, IL-6/JAK–STAT3, TNF-α/NF-κB, TGF-β/SMAD, CXCL/CXCR chemokine signaling, VEGF/VEGFR	Immune suppression, inflammatory remodeling, angiogenesis.	Breast cancer, prostate cancer, multiple myeloma, inflammatory and desmoplastic bone metastases	[13,61,62,63,64,65,68,70,71,72,73,74,76,110,118]
Mechanical ECM-remodeling axis	Perlecan/HSPG2, versican, biglycan, lumican, collagen-associated SLRPs, HA-rich matrix	LOX/LOXL2, MMP2/7/9/14, ADAMTS, cathepsins, collagen crosslinking enzymes	Integrins β1/β3–FAK/Src, YAP/TAZ, RhoA/ROCK, HIF-1α, PI3K/AKT	Stiffness, mechanotransduction, resistance.	Breast cancer, prostate cancer, desmoplastic and matrix-rich metastases	[15,20,75,105,117,137,143,168,182,183,184]
Translational PG/GAG biomarker and target axis	SDC1/CD138, HSPG2/perlecan fragments, HA-CD44/RHAMM, HPSE-modified HSPGs, decorin, PG signatures	HPSE, SULF1/2, HYAL1/2, MMPs, LOX/LOXL2	Matrix-remodeling signatures, circulating PGs/fragments, EV-associated PGs, liquid biopsy, niche-stratification markers	Biomarkers, stratification, therapeutic targeting.	Multiple myeloma, castration-resistant prostate cancer, breast cancer, matrix-rich tumors	[97,142,156,185,186,187,188,189,190]

Abbreviations: ADAMTS, a disintegrin and metalloproteinase with thrombospondin motifs; AKT, protein kinase B; BMP, bone morphogenetic protein; CAFs, cancer-associated fibroblasts; CD44, cluster of differentiation 44; CD138, cluster of differentiation 138/syndecan-1; RHAMM/CD168, receptor for hyaluronan-mediated motility; CCL2, C-C motif chemokine ligand 2; CCR2, C-C motif chemokine receptor 2; CXCL, C-X-C motif chewe confirm that the intended meaning has been carefully checked and has been retained.mokine ligand; CXCL12, C-X-C motif chemokine ligand 12; CXCR4, C-X-C motif chemokine receptor 4; ECM, extracellular matrix; EGF, epidermal growth factor; EGFR, epidermal growth factor receptor; EMT, epithelial–mesenchymal transition; ERBB2/HER2, erb-b2 receptor tyrosine kinase 2/human epidermal growth factor receptor 2; ERK, extracellular signal-regulated kinase; EVs, extracellular vesicles; FAK, focal adhesion kinase; FGF, fibroblast growth factor; FGFR, fibroblast growth factor receptor; GAG, glycosaminoglycan; GLI2, GLI family zinc finger 2; HA, hyaluronan; HAS2, hyaluronan synthase 2; HGF, hepatocyte growth factor; HPSE, heparanase; HS, heparan sulfate; HSPG, heparan sulfate proteoglycan; HSPG2, heparan sulfate proteoglycan 2/perlecan; HYAL, hyaluronidase; IGF, insulin-like growth factor; IGF1R, insulin-like growth factor 1 receptor; IL-6, interleukin-6; IL-8/CXCL8, interleukin-8/C-X-C motif chemokine ligand 8; JAK, Janus kinase; JNK, c-Jun N-terminal kinase; LOX/LOXL2, lysyl oxidase/lysyl oxidase-like 2; MET, mesenchymal–epithelial transition factor receptor; MMP, matrix metalloproteinase; MMP-7, matrix metalloproteinase 7; NF-κB, nuclear factor kappa B; OPG, osteoprotegerin; PDGF, platelet-derived growth factor; PDGFR, platelet-derived growth factor receptor; PG, proteoglycan; PI3K, phosphoinositide 3-kinase; PTHrP, parathyroid hormone-related protein; RANK, receptor activator of nuclear factor-κB; RANKL, receptor activator of nuclear factor-κB ligand; RB, retinoblastoma protein; ROCK, Rho-associated coiled-coil kinase; RTK, receptor tyrosine kinase; SDC, syndecan; SDC1, syndecan-1; Sema3A, semaphorin 3A; SHH, sonic hedgehog; SLRP, small leucine-rich proteoglycan; SMAD, suppressor of mothers against decapentaplegic proteins; Src, proto-oncogene tyrosine-protein kinase Src; STAT3, signal transducer and activator of transcription 3; SULF1/2, sulfatase 1/2; TAMs, tumor-associated macrophages; TGF-β, transforming growth factor beta; TGFβRIII, transforming growth factor beta receptor III/betaglycan; TLR, Toll-like receptor; VCAM-1, vascular cell adhesion molecule 1; VCAN, versican; VEGF, vascular endothelial growth factor; VEGFR, vascular endothelial growth factor receptor; WNT, wingless/integrated signaling pathway; YAP/TAZ, Yes-associated protein/transcriptional coactivator with PDZ-binding motif.

**Table 5:** Proteoglycan and GAG/ECM axes in breast cancer bone metastasis.

Axis	Main Bone-Metastasis Link	Experimental Model/Evidence Basis	Evidence Directness	Translational Relevance	Ref.
VCAN	Promotes motility, invasion, adhesion, EGFR/ERK–AKT signaling, and altered osteoblast behavior.	Breast cancer models including bone-metastasis/osteoblast-related studies.	Direct preclinical bone-metastasis/osteoblast-related evidence.	Prometastatic ECM marker; EGFR or ECM-remodeling pathways may be indirect targets.	[19,23,76,78,162]
HA-CD44/RHAMM axis	CD44, RHAMM/CD168, HAS2, ZEB1, ERK, PI3K/AKT	Breast cancer HA/CD44/HAS2 studies, including bone-progression-related models, plus broader HA-CD44/RHAMM cancer literature.	Mixed evidence. Direct bone-related evidence exists for selected HAS2/HA-CD44 models; RHAMM and broader HA-targeting claims are partly extrapolated.	Potential target in HA-rich aggressive niches; bone-specific validation remains limited.	[19,29,41,45,98,168,206]
Syndecans/HSPG co-receptors	Modulate HS-dependent growth-factor presentation, integrin signaling, FAK/Src activation, adhesion, and mechanotransduction.	Breast cancer progression studies and general mechanistic evidence for HSPG/syndecan-dependent signaling.	Mostly extrapolated for breast cancer skeletal metastasis. Bone-specific causal validation remains limited.	Candidate co-receptor axis; needs direct validation in breast cancer skeletal metastasis.	[19,42,94,134]
Perlecan/HSPG2	Acts as a growth-factor reservoir and regulates basement-membrane organization, angiogenesis, and tumor–stroma signaling.	TNBC and matrix-rich breast cancer models; broader HSPG2/perlecan and ECM-remodeling evidence	Extrapolated/bone-relevant but not breast bone-specific.	Promising matrix-associated target, but skeletal metastatic relevance remains incompletely validated.	[17,19,207]
DCN	Antagonizes EGFR, MET, HER2/ERBB2, VEGFR2, TGF-β, WNT/β-catenin, and angiogenic signaling; reduces osteolytic progression in preclinical models.	DCN-based breast cancer skeletal metastasis models and mechanistic RTK-inhibition studies.	Direct preclinical skeletal-metastasis evidence.	Strongest anti-metastatic PG candidate in this table; clinical translation still lacking.	[41,44,59,79,170]
SLRP/collagen-remodeling axis: biglycan, lumican and related SLRPs	Regulates collagen organization, inflammation, matrix stiffness, invasion, and context-dependent tumor suppression or promotion.	Breast cancer stromal studies; lumican bone-metastasis evidence is stronger in lung cancer models than in breast cancer.	Mostly extrapolated for breast cancer bone metastasis.	Useful as contextual matrix-state biomarkers; avoid presenting as proven breast bone-specific drivers.	[19,41,42,70]
SRGN	Associated with EMT, IL-8/CXCL8 signaling, protease secretion, inflammatory phenotype, and aggressive tumor behavior.	Breast cancer invasion and aggressiveness models.	Extrapolated. No direct skeletal-metastasis validation.	Potential marker of aggressive phenotype; role in skeletal metastasis remains unvalidated	[19,42,208]
Heparanase–HSPG remodeling	HPSE cleaves HS chains, releases matrix-bound growth factors, promotes angiogenesis, invasion, osteolytic remodeling, and tumor–bone vicious-cycle amplification.	HSPG/HPSE cancer models and osteolytic tumor-growth or bone-remodeling studies.	Bone-niche experimental/mixed evidence. Direct osteolytic relevance exists, but breast-specific skeletal validation remains incomplete.	Experimental target; may be relevant in combination with antiresorptive or tumor-directed therapy.	[17,22,94,207]

Evidence directness was classified as follows: Direct bone-metastasis evidence, when derived from patient bone metastasis samples or in vivo skeletal metastasis models; bone-niche experimental evidence, when derived from osteoblast, osteoclast, stromal, macrophage, mineralized matrix, or 3D bone-like models; and extrapolated evidence, when derived from primary tumors, non-bone metastatic sites, or general cancer models. Abbreviations: AKT, protein kinase B; CD44, cluster of differentiation 44; DCN, decorin; ECM, extracellular matrix; EGFR, epidermal growth factor receptor; EMT, epithelial–mesenchymal transition; ERBB2/HER2, erb-b2 receptor tyrosine kinase 2/human epidermal growth factor receptor 2; ERK, extracellular signal-regulated kinase; FAK, focal adhesion kinase; GAG, glycosaminoglycan; HA, hyaluronan; HAS2, hyaluronan synthase 2; HPSE, heparanase; HS, heparan sulfate; HSPG, heparan sulfate proteoglycan; HSPG2, heparan sulfate proteoglycan 2/perlecan; JNK, c-Jun N-terminal kinase; MET, mesenchymal–epithelial transition factor receptor; PI3K, phosphoinositide 3-kinase; RHAMM/CD168, receptor for hyaluronan-mediated motility; RTK, receptor tyrosine kinase; SDC, syndecan; SLRP, small leucine-rich proteoglycan; SRGN, serglycin; TNBC, triple-negative breast cancer; VCAN, versican; VEGFR2, vascular endothelial growth factor receptor 2; ZEB1, zinc finger E-box-binding homeobox 1.

**Table 6:** Proteoglycans and signaling axes in prostate cancer bone metastasis.

Proteoglycan/Axis	Main Mechanism in Prostate Cancer Bone Metastasis	Experimental Model/Evidence Basis	Evidence Directness	Translational Interpretation and Caution	Ref.
Perlecan/HSPG2	Supports desmoplastic matrix remodeling, growth-factor storage, angiogenesis, stromal invasion, and prostate cancer adaptation to the bone niche.	Prostate cancer bone-metastasis and desmoplastic microenvironment models; perlecan-rich matrix and tissue-invasion studies.	Direct preclinical/bone-relevant evidence.	Candidate biomarker of stromal invasion and matrix remodeling. HSPG2 is also linked to therapy resistance, including radioresistance, but clinical validation remains limited.	[16,26,28,123,131,132,133,161,212]
MMP-7–perlecan–Sema3A–FAK axis	MMP-7 cleavage of perlecan/Sema3A complexes activates FAK-dependent dyscohesion, migration, and stromal invasion.	Mechanistic prostate cancer matrix-invasion models and perlecan-fragment/tissue-invasion studies.	Direct mechanistic prostate bone-relevant evidence.	Strong mechanistic axis for matrix-dependent invasion. Therapeutic targeting remains preclinical; broad MMP or FAK inhibition may have toxicity and specificity limitations.	[20,123,156,161]
SULF1/SULF2–HSPG axis	Edits heparan sulfate sulfation, altering WNT3A, FGF, VEGF, and other ligand gradients in the bone niche.	3D prostate cancer–stroma–macrophage triculture models with perlecan-modified matrices; broader sulfatase/HSPG literature.	Bone-niche experimental evidence.	Potential modulator of HSPG-dependent signaling in desmoplastic and WNT-active niches. Context-dependent effects require caution; SULF1/SULF2 should not be described as uniformly pro- or anti-metastatic.	[17,27,35,103,113,175,176]
SDC1	Regulates adhesion, growth factor signaling, ectodomain shedding, invasion, and possible therapy resistance.	Advanced prostate cancer and CRPC biomarker studies; general syndecan/HSPG cancer mechanisms.	Mostly extrapolated for prostate cancer bone metastasis.	Circulating SDC1 has been associated with chemotherapy resistance in CRPC, but bone-specific validation is limited. Present as an exploratory biomarker, not as an established skeletal-metastasis marker.	[16,39,186]
Betaglycan/TGFβRIII	Mediates osteoblast-derived TGFβ2/GDF10 dormancy signals through p38MAPK–RB-dependent cell-cycle arrest.	Osteoblast-derived dormancy models and prostate cancer bone-niche studies.	Direct bone-niche dormancy evidence.	Key mechanistic axis for dormancy induction and maintenance. Translational targeting is not mature because disrupting dormancy signals could theoretically promote reactivation if not carefully controlled.	[82,83,84,179,180,181]
HA-CD44/RHAMM axis	Promotes motility, invasion, plasticity, androgen-independent progression, and adhesion to bone-niche components.	Prostate cancer progression models plus broader HA-CD44/RHAMM cancer studies.	Mixed/mostly extrapolated for skeletal metastasis.	Potential target in invasive or therapy-resistant subpopulations. HA is not a proteoglycan; it should be presented as an associated free GAG axis. Bone-specific therapeutic validation remains limited.	[16,29,31,99,163,168,213]
DCN	Suppresses tumor progression by antagonizing EGFR/AR, MET, VEGFR2, TGF-β-related signaling, angiogenesis, and invasion.	Preclinical prostate cancer models, including decorin-expressing oncolytic adenovirus studies in bone metastasis.	Direct preclinical skeletal-metastasis evidence.	Promising anti-metastatic strategy, but still preclinical. Delivery, immune response, off-target matrix effects, and clinical efficacy remain unresolved.	[16,24,44,59,171]
VCAN	May promote ECM remodeling, inflammatory signaling, invasion, and tumor-cell plasticity in a context-dependent manner.	Prostate cancer and ECM-remodeling literature; stronger mechanistic support comes from broader cancer and matrix studies.	Mostly extrapolated for prostate cancer bone metastasis.	Potential marker of a matrix-remodeled niche, but not a validated prostate skeletal-metastasis biomarker. Use cautious wording.	[16,42,76,110]
Therapy-resistance-associated matrix PGs: ASPN, BGN, SPOCK1/testican-1	Associated with inflammatory stroma, EMT/plasticity, CRPC progression, and resistance to systemic therapies.	Advanced prostate cancer and therapy-resistance studies; evidence not consistently derived from skeletal metastasis.	Extrapolated for bone metastasis.	Exploratory biomarkers of aggressive or resistant disease. Avoid implying bone-specific predictive value until validated in skeletal metastasis samples or bone-niche models.	[16,41]
Heparanase/HPSE–HSPG axis	Cleaves HS chains, releases matrix-bound growth factors, and promotes ECM remodeling, angiogenesis, invasion, and mixed bone remodeling.	HSPG/heparanase cancer models, osteolytic tumor growth studies, and broader evidence of prostate/bone matrix remodeling.	Mixed evidence. Bone-relevant preclinical evidence exists, but prostate bone-specific validation is incomplete.	HPSE inhibitors are investigational. Potential use may be in combination with systemic therapy, antiresorptive agents, or ECM-targeted approaches; toxicity and broad ECM effects require caution.	[17,22,34,102,146,191,192]

Abbreviations: 3D, three-dimensional; AKT, protein kinase B; AR, androgen receptor; ASPN, asporin; BGN, biglycan; CD44, cluster of differentiation 44; RHAMM/CD168, receptor for hyaluronan-mediated motility; CRPC, castration-resistant prostate cancer; DCN, decorin; ECM, extracellular matrix; EGFR, epidermal growth factor receptor; ERK, extracellular signal-regulated kinase; FAK, focal adhesion kinase; FGF, fibroblast growth factor; GDF10, growth differentiation factor 10; HA, hyaluronan; HGF, hepatocyte growth factor; HPSE, heparanase; HS, heparan sulfate; HSPG, heparan sulfate proteoglycan; HSPG2, heparan sulfate proteoglycan 2/perlecan; MET, mesenchymal–epithelial transition factor receptor; MMP-7, matrix metalloproteinase 7; NF-κB, nuclear factor kappa B; PI3K, phosphoinositide 3-kinase; RB, retinoblastoma protein; ROCK1, Rho-associated coiled-coil kinase 1; RTK, receptor tyrosine kinase; SDC1, syndecan-1; Sema3A, semaphorin 3A; Src, proto-oncogene tyrosine-protein kinase Src; SULF1/2, sulfatase 1/2; TGF-β, transforming growth factor beta; TGFβRIII, transforming growth factor beta receptor III/betaglycan; TLR, Toll-like receptor; VCAN, versican; VEGF, vascular endothelial growth factor; VEGFR2, vascular endothelial growth factor receptor 2; WNT, wingless/integrated signaling pathway. Evidence directness: Direct bone-metastasis evidence, evidence derived from patient bone metastasis samples or in vivo skeletal metastasis models; bone-niche experimental evidence, evidence derived from osteoblast, osteoclast, stromal, macrophage, mineralized matrix, or 3D bone-like models; extrapolated evidence, evidence derived mainly from primary tumors, non-bone metastatic sites, or general cancer models.

**Table 8:** Knowledge gaps and future directions for proteoglycan research in bone metastasis.

Knowledge Gap	Limitation	Priority	Impact	Ref.
Spatial mapping of the metastatic bone matrix	PGs are usually studied individually, without spatial integration of core proteins, GAGs, sulfation, fragments, cell source, and lesion phenotype.	Build spatial PG/GAG maps using matrisomics, spatial proteomics, spatial transcriptomics, GAGomics, degradomics, and insoluble matrix proteomics.	Define matrix-state signatures for osteolytic, osteoblastic, mixed, dormant, inflammatory, and resistant niches.	[182,198,199,200,201,202,203,204]
Human bone metastasis validation	Human bone biopsies are scarce, difficult to process, and affected by decalcification; most evidence comes from models.	Optimize workflows for fresh, frozen, FFPE, and decalcified bone metastasis samples.	Improve clinical relevance of PG signatures and identify their cellular origin in human lesions.	[19,48,138,195,196]
Association versus causality	Many studies are correlative and do not prove whether PGs drive colonization, dormancy, immune escape, or resistance.	Use gain- and loss-of-function studies in multicellular mineralized bone niche models.	Prioritize causal PG-dependent mechanisms as therapeutic targets.	[37,42,70,195,196,236]
PG–immune interface	The role of PG/GAG remodeling in immune-cell positioning, exclusion, activation, or suppression remains unclear.	Combine spatial immune profiling with PG/GAG mapping in bone metastases.	Support matrix-informed immunotherapy combinations.	[62,63,65,70,71,72,73,75,76,143,159]
Dormancy and reactivation	How matrix stiffness, GAG sulfation, PG fragmentation, HPSE, HA-CD44/RHAMM, and osteoclast remodeling regulate reactivation is unresolved.	Model dormant and reactivated tumor cells in endosteal and marrow-like niches.	Identify biomarkers and interventions to prevent late skeletal relapse.	[80,81,82,83,84,86,101,121,179,180,181]
Niche-informed precision medicine	Current stratification relies primarily on tumor lineage, imaging, bone turnover markers, and systemic therapy response.	Integrate imaging, serum markers, circulating PG fragments, EV cargo, ECM signatures, immune profiling, and tumor genomics.	Classify patients by niche state, matrix remodeling, immune context, dormancy risk, and treatment vulnerability.	[22,31,106,142,143,183,185,190,229,230]
Context-specific targeting	Broad PG or enzyme inhibition may affect normal bone, vascular, hematopoietic, and immune functions.	Target HPSE, SULF1/2, HA-CD44/RHAMM, MMPs, cathepsins, LOX/LOXL2, integrin–FAK/Src, and decorin-regulated RTK pathways selectively.	Reprogram the metastatic niche to reduce colonization, immune suppression, reactivation, and therapy resistance.	[15,22,31,35,102,103,142,143,144,175,182,183,184,190,229,230]

Abbreviations: ECM, extracellular matrix; EV, extracellular vesicle; FAK, focal adhesion kinase; FFPE, formalin-fixed paraffin-embedded; GAG, glycosaminoglycan; HA, hyaluronan; HPSE, heparanase; LOX/LOXL2, lysyl oxidase/lysyl oxidase-like 2; MMP, matrix metalloproteinase; PG, proteoglycan; RHAMM, receptor for hyaluronan-mediated motility; RTK, receptor tyrosine kinase; Src, proto-oncogene tyrosine-protein kinase Src; SULF1/2, sulfatase 1/2.

## Data Availability

No new data were created or analyzed in this study. Data sharing is not applicable to this article.

## Abbreviations

The following abbreviations are used in this manuscript:

ADAMTS	A disintegrin and metalloproteinase with thrombospondin motifs
AKT	Protein kinase B
AR	Androgen receptor
ARG1	Arginase 1
ASPN	Asporin
BGN	Biglycan
BMP	Bone morphogenetic protein
CAFs	Cancer-associated fibroblasts
cAMP	Cyclic adenosine monophosphate
CCL	C-C motif chemokine ligand
CCR	C-C motif chemokine receptor
CD	Cluster of differentiation
CD1d	Cluster of differentiation 1d
CD44	Cluster of differentiation 44
RHAMM/CD168	Receptor for hyaluronan-mediated motility
CREB	cAMP response element-binding protein
CSF-1	Colony-stimulating factor 1
CSF-1R	Colony-stimulating factor 1 receptor
CSPG4	Chondroitin sulfate proteoglycan 4
CTLA-4	Cytotoxic T-lymphocyte-associated protein 4
CXCL	C-X-C motif chemokine ligand
CXCR	C-X-C motif chemokine receptor
DCN	Decorin
DCs	Dendritic cells
DNAM-1	DNAX accessory molecule 1
ECM	Extracellular matrix
EGF	Epidermal growth factor
EGFR	Epidermal growth factor receptor
eIF4E3	Eukaryotic translation initiation factor 4E family member 3
EMT	Epithelial–mesenchymal transition
ERBB2/HER2	Erb-b2 receptor tyrosine kinase 2/human epidermal growth factor receptor 2
ERK	Extracellular signal-regulated kinase
FAK	Focal adhesion kinase
FGF	Fibroblast growth factor
FGFR	Fibroblast growth factor receptor
FOXP3	Forkhead box P3
Gab1	GRB2-associated-binding protein 1
GAG	Glycosaminoglycan
GDF10	Growth differentiation factor 10
GLI2	GLI family zinc finger 2
GPC3	Glypican-3
GPC6	Glypican-6
GPI	Glycosylphosphatidylinositol
HA	Hyaluronan
HAS2	Hyaluronan synthase 2
HGF	Hepatocyte growth factor
HPSE	Heparanase
HSPG	Heparan sulfate proteoglycan
HSPG2	Heparan sulfate proteoglycan 2/perlecan
HSPGs	Heparan sulfate proteoglycans
IFN-γ	Interferon gamma
IGF	Insulin-like growth factor
IGF1R	Insulin-like growth factor 1 receptor
IL	Interleukin
IL-8/CXCL8	Interleukin-8/C-X-C motif chemokine ligand 8
iNOS	Inducible nitric oxide synthase
JAK	Janus kinase
JNK	C-Jun N-terminal kinase
LOX/LOXL2	Lysyl oxidase/lysyl oxidase-like 2
MAMs	Metastasis-associated macrophages
MAPK	Mitogen-activated protein kinase
MDSCs	Myeloid-derived suppressor cells
MET	Mesenchymal–epithelial transition factor receptor
MFG-E8	Milk fat globule-EGF factor 8
MHC	Major histocompatibility complex
MMP	Matrix metalloproteinase
MMP-7	Matrix metalloproteinase 7
MMPs	Matrix metalloproteinases
NF-κB	Nuclear factor kappa B
NG2	Neuron-glial antigen 2
NK cells	Natural killer cells
NKT cells	Natural killer T cells
NO	Nitric oxide
OPG	Osteoprotegerin
PD-1	Programmed cell death protein 1
PD-L1	Programmed death-ligand 1
PDGF	Platelet-derived growth factor
PDGFR	Platelet-derived growth factor receptor
PG	Proteoglycan
PGE2	Prostaglandin E2
PI3K	Phosphoinositide 3-kinase
PKA	Protein kinase A
PKCα	Protein kinase C alpha
PTHrP	Parathyroid hormone-related protein
p38 MAPK	p38 mitogen-activated protein kinase
RANK	Receptor activator of nuclear factor-κB
RANKL	Receptor activator of nuclear factor-κB ligand
RB	Retinoblastoma protein
RHAMM	Receptor for hyaluronan-mediated motility
Rho GTPase	Rho guanosine triphosphatase
ROCK	Rho-associated coiled-coil kinase
ROCK1	Rho-associated coiled-coil kinase 1
ROS	Reactive oxygen species
RTK	Receptor tyrosine kinase
scRNA-seq	Single-cell RNA sequencing
SDC	Syndecan
SDC1	Syndecan-1
SDC1/CD138	Syndecan-1/cluster of differentiation 138
Sema3A	Semaphorin 3A
SHH	Sonic hedgehog
SLRP	Small leucine-rich proteoglycan
SMAD	Suppressor of mothers against decapentaplegic proteins
Src	Proto-oncogene tyrosine-protein kinase Src
SREs	Skeletal-related events
SRGN	Serglycin
STAT3	Signal transducer and activator of transcription 3
SULF1/2	Sulfatase 1/2
TAMs	Tumor-associated macrophages
TANs	Tumor-associated neutrophils
Tc17	IL-17-producing cytotoxic T cells
TGF-β	Transforming growth factor beta
TGFβ-RIII	Transforming growth factor beta receptor III/betaglycan
Th	T helper cell
TLR	Toll-like receptor
Tregs	Regulatory T cells
VCAM-1	Vascular cell adhesion molecule 1
VCAN	Versican
VEGFA	Vascular endothelial growth factor A
VEGF	Vascular endothelial growth factor
VEGFR	Vascular endothelial growth factor receptor
VEGFR2	Vascular endothelial growth factor receptor 2
WNT	Wingless/integrated signaling pathway
WNT3A	Wingless-type MMTV integration site family member 3A
YAP/TAZ	Yes-associated protein/transcriptional coactivator with PDZ-binding motif
ZEB1	Zinc finger E-box-binding homeobox 1
